# Characterisation of the Stromal Microenvironment in Lobular Breast Cancer

**DOI:** 10.3390/cancers14040904

**Published:** 2022-02-11

**Authors:** Laura Gómez-Cuadrado, Esme Bullock, Zeanap Mabruk, Hong Zhao, Margarita Souleimanova, Pernille Rimmer Noer, Arran K. Turnbull, Claus Oxvig, Nicholas Bertos, Adam Byron, J. Michael Dixon, Morag Park, Syed Haider, Rachael Natrajan, Andrew H. Sims, Valerie G. Brunton

**Affiliations:** 1 Cancer Research UK Edinburgh Centre, Institute of Genetics and Cancer, University of Edinburgh, Crewe Road South, Edinburgh EH4 2XR, UK; laura.gomezcuadrado@gmail.com (L.G.-C.); esme.bullock@ed.ac.uk (E.B.); z.mabruk@sms.ed.ac.uk (Z.M.); a.turnbull@ed.ac.uk (A.K.T.); adam.byron@ed.ac.uk (A.B.); 2 Goodman Cancer Research Centre, McGill University, Montreal, QC H3A 1A3, Canada; hong.zhao@mcgill.ca (H.Z.); margarita.souleimanova@mcgill.ca (M.S.); morag.park@mcgill.ca (M.P.); 3 Department of Molecular Biology and Genetics, University of Aarhus, DK-8000 Aarhus C, Denmark; pernille.noer@mbg.au.dk (P.R.N.); co@mbg.au.dk (C.O.); 4 Research Institute of the McGill University Health Centre, Montreal, QC H4A 3J1, Canada; nicholas.bertos@mail.mcgill.ca; 5 Edinburgh Breast Unit, University of Edinburgh, Edinburgh EH4 2XU, UK; mike.dixon@ed.ac.uk; 6 The Breast Cancer Now Toby Robins Research Centre, The Institute of Cancer Research, London SW3 6JB, UK; syed.haider@icr.ac.uk (S.H.); rachael.natrajan@icr.ac.uk (R.N.)

**Keywords:** lobular breast cancer, tumor microenvironment, cancer-associated fibroblasts

## Abstract

**Simple Summary:**

Invasive lobular breast cancer (ILC) accounts for approximately 5–15% of breast cancers, and although response rates to treatments are initially good, an ILC diagnosis is associated with adverse long-term outcomes; better treatments, specifically targeted to this sub-type of breast cancer, are required to improve patient survival. The tumor microenvironment (TME) plays an important role in determining how cancers respond to treatment, and in this study, we carried out an in-depth analysis of the TME in ILC following laser-capture microdissection of the tumor stroma, and analysis of primary cancer-associated fibroblasts (CAFs), which comprise the majority of non-malignant cells within tumors. This identified changes in genes involved in regulation of the extracellular matrix and also growth factor signaling pathways that were differentially regulated in ILC. Further analysis of breast cancer datasets showed that two of these genes which encode a secreted metalloproteinase (*PAPPA*) and a metalloproteinase inhibitor (*TIMP2*) were associated with survival outcomes in ILC.

**Abstract:**

Invasive lobular carcinoma (ILC) is the second most common histological subtype of breast cancer, and it exhibits a number of clinico-pathological characteristics distinct from the more common invasive ductal carcinoma (IDC). We set out to identify alterations in the tumor microenvironment (TME) of ILC. We used laser-capture microdissection to separate tumor epithelium from stroma in 23 ER+ ILC primary tumors. Gene expression analysis identified 45 genes involved in regulation of the extracellular matrix (ECM) that were enriched in the non-immune stroma of ILC, but not in non-immune stroma from ER+ IDC or normal breast. Of these, 10 were expressed in cancer-associated fibroblasts (CAFs) and were increased in ILC compared to IDC in bulk gene expression datasets, with *PAPPA* and *TIMP2* being associated with better survival in ILC but not IDC. *PAPPA,* a gene involved in IGF-1 signaling, was the most enriched in the stroma compared to the tumor epithelial compartment in ILC. Analysis of *PAPPA*- and *IGF1*-associated genes identified a paracrine signaling pathway, and active PAPP-A was shown to be secreted from primary CAFs. This is the first study to demonstrate molecular differences in the TME between ILC and IDC identifying differences in matrix organization and growth factor signaling pathways.

## 1. Introduction

Invasive lobular breast cancer (ILC) accounts for approximately 5–15% of breast cancers and is the second most common histological subtype after invasive breast cancer of no specific type, commonly referred to as invasive ductal carcinoma (IDC). ILC is recognized to exhibit a number of clinico-pathological characteristics distinct from those of IDC [1,2]. It has an increased propensity for multi-centricity, multi-focality and bilaterality, in addition to an unusual pattern of metastatic dissemination [3]. ILC is predominantly estrogen receptor (ER)- and progesterone receptor-positive, with low to absent expression of human epidermal growth factor receptor-2. Most patients with ILC are candidates for adjuvant endocrine treatment. Although response rates are initially good, an ILC diagnosis is associated with adverse long-term outcomes compared to IDC [4]. At the molecular level, ILC is defined by a loss or reduced expression of E-cadherin, and several studies have further mapped the genomic landscape of ILC [5,6,7,8,9]. More recently, tumor-infiltrating lymphocyte populations have also been profiled [10]. ILC is characterized by having a dense stroma with a larger contact area with tumor cells than IDC, due in part to the difference in tumor growth pattern (single file versus dense islands, respectively). However, little is known about the composition of the stroma or the role of the surrounding tumor microenvironment (TME). The TME plays a critical role in tumor behavior by influencing progression and metastatic spread, as well as therapeutic response [11,12], and in breast cancer, a stroma-derived prognostic predictor has been identified that stratifies disease outcome independently of clinical prognostic factors [13].

In this study, we used laser-capture microdissection (LCM) of human ILC, combined with analysis of primary patient-derived cancer associated fibroblasts (CAFs) from both ILC and IDC, to generate the first in-depth transcriptomic characterization of human ILC stroma. This identified significant differences in stromal gene signatures in ILC compared to IDC: a number of genes involved in ECM regulation were more highly expressed in the stroma compared to the tumor epithelium in ILC, but not in IDC or normal breast. Comprehensive survival analysis of stromal ILC genes in human breast cancer datasets identified a positive association with improved survival and *PAPPA* and *TIMP2* (tissue inhibitor of metalloproteinase 2) in ILC but not IDC. *PAPPA* was the most enriched gene in the stroma compared to the tumor epithelial compartment in ILC. *PAPPA* encodes pregnancy-associated plasma protein-A (PAPP-A), a metalloproteinase that cleaves insulin-like growth factor-binding protein-4 (IGFBP-4), increasing IGF-1 bioavailability and downstream signaling. Analysis of *PAPPA*- and *IGF1*-associated genes identified a paracrine signaling pathway, and active PAPP-A was shown to be secreted from primary CAFs. This study provides a detailed analysis of the TME in ILC and highlights differences in both matrix regulatory and growth factor signaling pathways.

## 2. Materials and Methods

All standard assays not detailed here are described in the Supplementary Methods (available online).

### 2.1. Tissue Processing for LCM and Gene Expression Analysis

All samples were obtained from the McGill University Health Centre: Breast Cancer Functional Genomics Initiative Biobank, Montreal, Canada (study identifiers SUR-99-780 and SUR-2000-966). Prior to LCM, tumor tissues were stained for epithelial (cytokeratin) and immune (CD45) markers, and an adjacent section was stained with H&E to visualize the tissue morphology and the different cell types present in the sample. Tumor epithelial cells were microdissected from an adjacent section based on the morphology (round), growth pattern (indian file) and cytokeratin (CK18) staining. Tumor-associated stromal tissue was isolated by microdissecting CAFs (based on their spindle morphology) and matrix surrounding the CAFs. Immune cells were excluded during the laser-capture microdissection; they were distinguished from the rest of stromal cells based on CD45 positive staining, as well as their characteristic morphology on the H&E slide. Subsequent LCM, RNA extraction and microarray hybridization were carried out as previously described [14] and analyzed using the SurePrint G3 Human GE 8 × 60 K microarray kit (Supplementary Figure S1). Processed and raw data are available from Gene Expression Omnibus (GSE148398).

### 2.2. Primary CAF RNA-Seq Dataset Generation

All samples were obtained from the NHS Lothian Tissue Governance Committee, Edinburgh, United Kingdom (approval number 15/ES/0094). CAFs were isolated from eleven ILC and five IDC samples, and total RNA was extracted (Qiagen) from three biological repeats of each sample except ED2334 CAFs where two repeats were available. The Lexogen QuantSeq 3′mRNA-Seq Library Prep kit (FWD) was used for library generation and single-read sequencing was carried out on the Illumina NextSeq 550 platform. Sequence alignment and counting was performed using the QuantSeq 3′mRNA pipeline on the Bluebee platform. The data were normalized by trimmed mean of M-values (TMM) [15], and differential gene expression analysis (ILC v IDC CAFs) was performed using a generalized linear model (GLM) likelihood ratio test in EdgeR R package [16]. FDR < 0.05 was considered significant.

### 2.3. Gene Set Enrichment Analysis

GSEA was run in GSEA_4.1.0 desktop application with geneset permutation. The molecular signature database (MSigDB) Hallmarks genesets [17] were analyzed, and FDR < 0.05 was considered significant [18].

### 2.4. Statistical Analysis

All statistical analyses were two-sided, and *p* < 0.05 was considered statistically significant. Differential gene expression analyses from the LCM dataset were calculated using rank products in MeV [19]. Differences in gene expression in whole tissues were assessed by Wilcoxon test, in primary CAFs by Mann–Whitney–Wilcoxon test and GLM likelihood ratio, and in KEP tumor and CAFs by *t*-test. Mann–Whitney–Wilcoxon test and fold change analysis were used to assess differences in gene expression between tumor and stroma in LCM datasets. Correlation between *PAPPA* and *IGF1* in LCM datasets and *PAPPA* and *IGF1* pathway genes and phospho-IGFR1 in the TCGA datasets was assessed by Pearson correlation and linear regression analysis in Graphpad Prism. Gene expression and survival analyses were performed using the R statistical programming environment. For survival analysis, preprocessed publicly available datasets were used (TCGA BRCA [20], METABRIC [21], Desmedt/GSE88770 [10] and SCAN-B [22]).

## 3. Results

### 3.1. Generation of an LCM-ILC Dataset 

**Figure 1:**
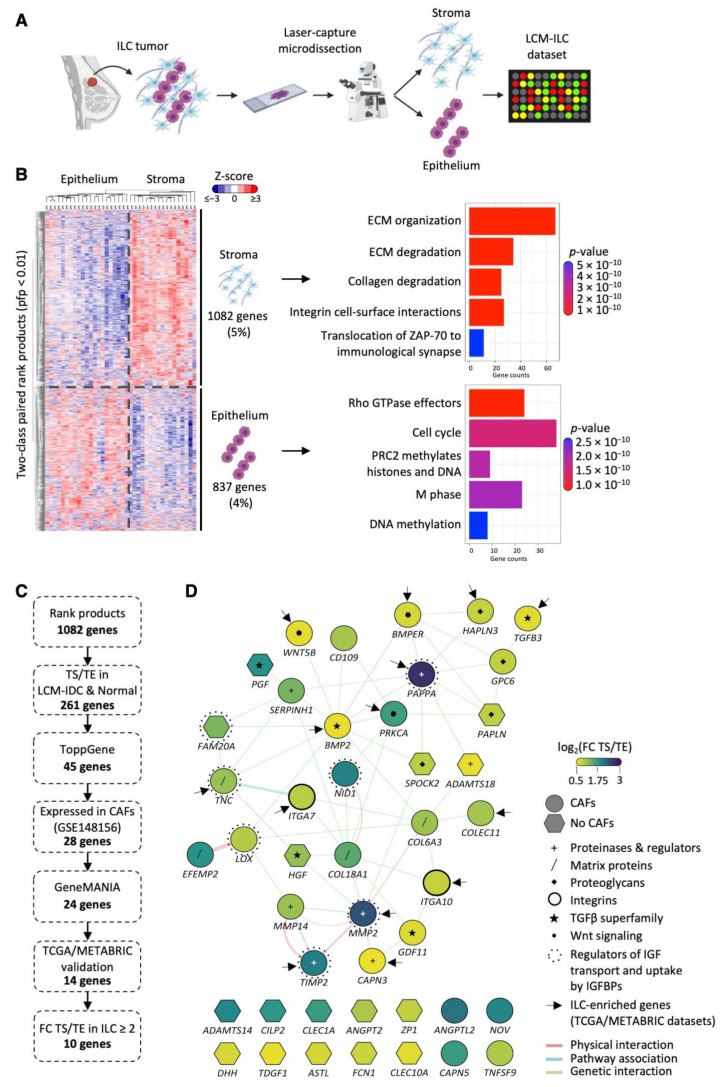
Identification of stromal genes enriched in ILC following LCM. (**A**) Schematic representation of the LCM experimental design (made in Biorender.com (accessed on 1 June 2020)). (**B**) Two-class paired differential gene expression analysis and Reactome pathway enrichment analysis of 22 tumor epithelial (TE) and 18 tumor stromal (TS) ILC samples dissected by LCM. Heatmap was generated using MultiExperiment Viewer (rank products, pfp < 0.01), Cluster 3.0 and TreeView. Z-scored expression values are shown. Reactome bar plot was generated in R. (**C**) Pipeline used for the selection of TS-ILC-enriched genes from the LCM-ILC dataset. (**D**) Interaction map of the 45 ECM-associated genes from ToppGene was generated using GeneMANIA in Cytoscape. Color represents the log_2_(fold change (FC)) expression in the TS compared to TE. Purple, log_2_(FC TS/TE) 3; green, log_2_(FC TS/TE) 1.75; yellow, log_2_(FC TS/TE) 0.5. Network connectors represent physical interactions (pink), pathway associations (blue) or genetic interactions (green). Arrows point to the 14 genes whose expression was significantly increased in ER+ ILC compared to ER+ IDC in TCGA and METABRIC datasets.

LCM was performed on 23 ILC fresh frozen human samples: 17 were Grade 2 (74%), five Grade 1 and one Grade 3. RNA was isolated from tumor epithelium (TE) and tumor stroma (TS) compartments. As recent studies have mapped the immune landscape in ILC, here we wanted to focus on gene expression in the CAFs. TS is therefore defined as primarily CAFs and matrix proteins, with the majority of immune cells being excluded (see Materials and Methods) to enhance the purity of the CAFs. Gene expression data were generated for a total of 22 TE and 18 TS samples (Figure 1A; Supplementary Figure S1), including matched pairs from 17 samples. Two-class paired rank product analysis (percent false positive (pfp) < 0.01) identified 1082 genes significantly highly expressed in the TS and 837 in the TE. These genes clustered the samples by compartment type (epithelium/stroma), showing successful microdissection of TE and TS compartments. Biomolecular pathway annotation revealed upregulation of genes involved in extracellular matrix (ECM) remodeling, collagen degradation and integrin cell surface interactions in TS compared to TE, while genes related to cell cycle, DNA replication and methylation were upregulated in TE compared to TS compartments (Figure 1B).

### 3.2. Identification of TS-ILC Enriched Genes

An analysis pipeline was set up to identify genes upregulated in the TS compared to TE in ILC, but not IDC or normal breast (Figure 1C). First, the list of 1082 genes differentially expressed in our TS LCM-ILC dataset was applied to previously reported LCM-IDC (GSE68744) [23] and LCM-normal (GSE4823) [14] datasets. This identified 261 genes increased in the TS compared to TE only in ILC. Pathway enrichment analysis (https://toppgene.cchmc.org/ (accessed on 1 November 2019)) identified 45 of these genes to be involved in significantly over-represented pathways (Benjamini–Hochberg adjusted *p*-value < 0.05), all related to the ECM (Supplementary Table S1). Network analysis revealed 30 interconnected genes (Figure 1D), including matrix proteins (*COL6A3, COL18A1, TNC*, *EFEMP2*), proteoglycans (*SPOCK2*, *PAPLN*, *HAPLN3*, *GPC6*), proteinases and their regulators (*MMP2*, *TIMP2*, *MMP14*, *CAPN3*, *ADAMTS18*, *SERPINH1*, *PAPPA*), and integrin subunits (*ITGA7, ITGA10*). A number of growth factors (*PGF*, *HGF*), including those of the TGFβ superfamily (*GFD11*, *TGFB3*, *BMP2*), were also identified. The analysis highlighted physical interactions of MMP2 with *TIMP2*, *MMP14*, *COL6A3*, *COL18A1* and *CAPN3* gene products, all involved in ECM organization. In addition, *BMP2* and *PAPPA* were the two main hubs of genetic interactions [24] (Figure 1D). The expression of these 45 stromal genes was examined in ILC and IDC ER+ samples from the METABRIC [21] and The Cancer Genome Atlas (TCGA; http://cancergenome.nih.gov/ (accessed on 1 November 2019)) bulk mRNA datasets (Table 1 and Supplementary Table S2). *PAPPA*, *PRKCA*, *TGFB3*, *ITGA10*, *ITGA7*, *CLEC1A*, *CLEC10A* and *PAPLN* were upregulated in ILC compared to IDC in both the METABRIC and TCGA datasets, highlighting the importance of these genes in the stroma of lobular carcinoma (Supplementary Figure S2).

**Figure 2:**
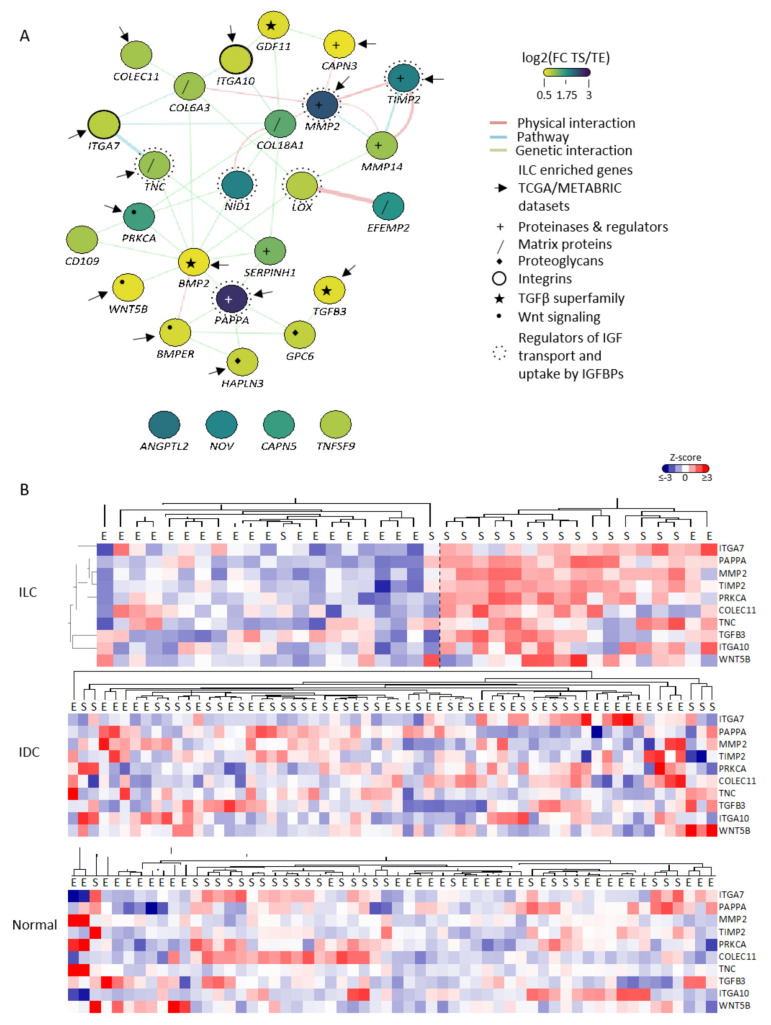
CAF-associated genes identified in the LCM-ILC dataset are highly connected and enriched in the stroma of ILC. (**A**) Interaction map of the 28 CAF-associated genes identified in the LCM-ILC dataset was generated using GeneMANIA in Cytoscape. Color represents the log2(fold change (FC)) expression in the TS compared to TE. Purple, log_2_(FC TS/TE) 3; green, log_2_(FC TS/TE) 1.75; yellow, log_2_(FC TS/TE) 0.5. Network connectors represent physical interactions (pink), pathway associations (blue) or genetic interactions (green). Arrows point to the 14 genes whose expression was significantly increased in ILC compared to IDC in TCGA and METABRIC datasets. (**B**) Heatmaps of CAF-associated genes in ILC, IDC- (GSE68744) and normal- (GSE4823) LCM datasets were generated from Z-scored values using Cluster 3.0 and TreeView. Tissue samples are indicated as E = epithelium, S = stroma.

Of the 45 ILC-specific stromal genes identified, 28 were expressed in CAFs (GSE148156). Functional network analysis (http://genemania.org/ (accessed on 1 November 2019)) identified that 24 of these 28 genes are in the same pathway or are linked by known genetic or physical interactions (Figure 2A). The majority (14/24) were significantly upregulated in ILC compared to IDC (*p* < 0.05) in at least one of the published bulk datasets (Table 1). Clustering the 10 genes with a TS/TE fold change > 2 in the LCM-ILC dataset clearly showed increased expression in the TS of ILC, but not in IDC or normal breast (Figure 2B).

### 3.3. ILC-Specific Gene Expression in Primary Cancer-Associated Fibroblasts

**Figure 3:**
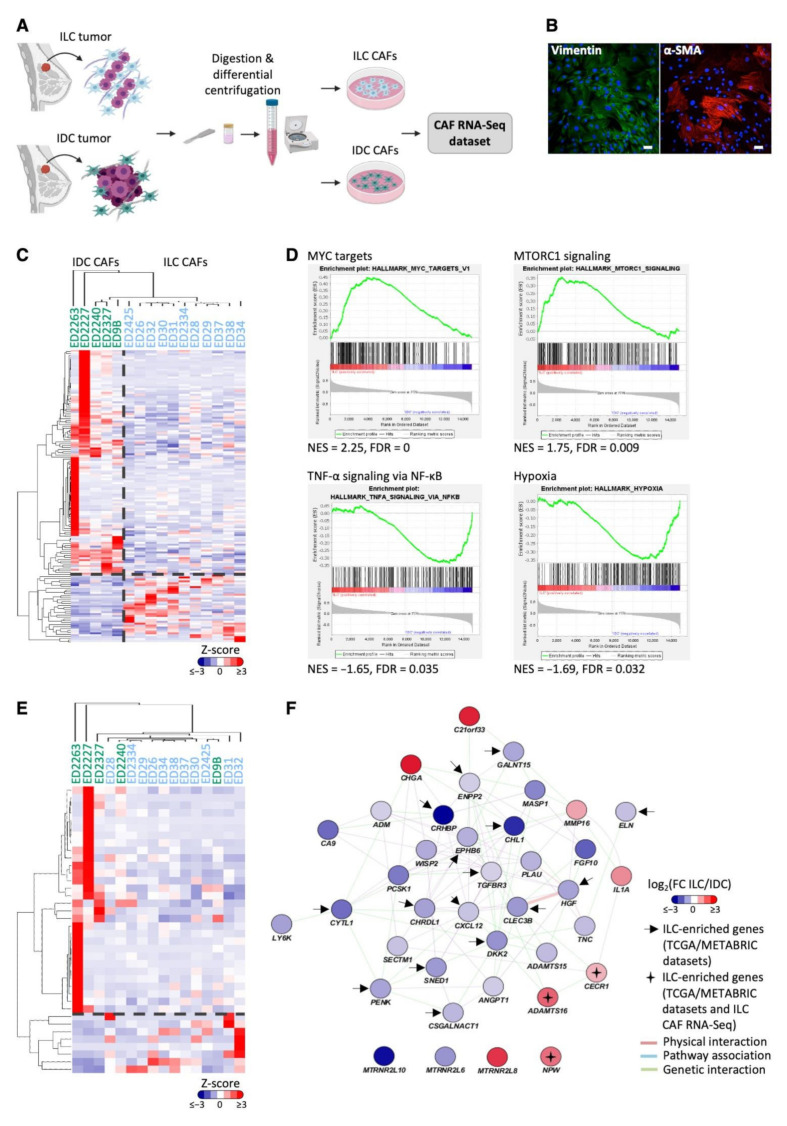
Generation of a primary CAF dataset identifies genes and pathways specifically enriched in either ILC or IDC CAFs. (**A**) Schematic representation of primary CAF dataset generation made in Biorender.com (accessed on 1 June 2020). (**B**) Primary ED2425 ILC CAFs stained for the mesenchymal markers vimentin (green) and α-SMA (red). Scale bar, 50 μm. (**C**) Differential gene expression between ILC and IDC CAFs determined by likelihood ratio test (EdgeR, glmFit). Heatmap of significantly differentially expressed genes (FDR < 0.05) was generated using Cluster 3.0 and TreeView. Z-scored expression values are shown. (**D**) Gene set enrichment analysis of ILC and IDC CAF gene expression. Significant hallmark gene sets FDR < 0.05. (**E**) Heatmap of significantly differentially expressed secreted genes (FDR < 0.05) between ILC and IDC CAFs determined by likelihood ratio test (EdgeR, glmFit) generated using Cluster 3.0 and TreeView. Z-scored expression values are shown. (**F**) GeneMANIA network created in Cytoscape showing the 38 significantly differentially expressed secreted protein encoding genes between ILC and IDC CAFs in the RNA-Seq dataset. Positive log fold-change shows higher expression in ILC CAFs (in red) and negative log fold-change shows higher expression in IDC CAFs (blue). Arrows indicate genes upregulated in ILC tumors compared to IDC tumors in 2 out of 3 bulk tumor datasets (METABRIC, TCGA RNA-Seq and Microarray datasets), with crosses indicating genes upregulated in both ILC CAFs and tumors. Network connectors represent physical interactions (pink), pathway associations (blue) or genetic interactions (green).

To directly compare CAFs from ILC and IDC tumors, we carried out RNA-Seq analysis on primary CAFs from 11 ILC and 5 IDC tumors (Figure 3A,B; Supplementary Figure S3). One-hundred-and-fifty-three genes were found to be differentially expressed between ILC and IDC CAFs (FDR < 0.05); 36 of these genes were upregulated in ILC CAFs compared to IDC CAFs (Figure 3C). The differentially expressed genes were enriched for ECM-associated genes (ToppGene, FDR B&Y < 0.05), and gene set enrichment analysis (GSEA) revealed that MYC targets and mTORC signaling gene sets were significantly enriched in ILC CAFs, whereas IDC CAFs were enriched for TNFα signaling and hypoxia gene sets (FDR < 0.05, Figure 3D).

Proteins secreted by CAFs can act in a paracrine manner to induce signaling in tumor epithelial cells and may represent potentially interesting and therapeutically accessible targets. The CAF RNA-Seq dataset was filtered for genes encoding secreted proteins [25], leaving 1536 genes encoding predicted secreted proteins expressed in ILC and IDC CAFs. Thirty-eight genes were identified as differentially expressed between ILC and IDC CAFs, of which eight were upregulated in ILC CAFs (Figure 3E). Network analysis identified genetic interactions between *ADAMTS16* and *CECR1* as well as *MMP16* and *IL1A* [24] and showed co-expression of *MMP16*, *IL1A* and *CECR1* (Figure 3F). *ADAMTS16*, *CECR1* and *NPW* were also significantly more highly expressed in ILC tumors compared to IDC ER+ tumors in the TCGA dataset (Table 2).

Of the 261 genes identified in the LCM dataset to be increased in the TS compared to TE only in ILC (Figure 1), 186 of these were expressed by primary ILC and IDC CAFs in the RNA-Seq dataset. Of these, 27 were significantly differentially expressed between ILC and IDC CAFs. Functional network analysis (http://genemania.org/ (accessed on 1 June 2021)) identified that 18 of these 27 genes are linked by known genetic or physical interactions (Supplementary Figure S4A), with 10 showing significantly higher expression in the ILC CAFs (Supplementary Figure S4B; Supplementary Table S3). The expression of these 10 genes was examined in ILC and IDC ER+ samples from the METABRIC and TCGA bulk mRNA datasets (Supplementary Table S3): *CECR1, AKT3*, *EDA2R* and *MYH3* were upregulated in ILC compared to IDC in TCGA dataset, with *AASS* and *MBP* being upregulated in both the METABRIC and TCGA datasets.

### 3.4. Survival Analysis of CAF-Associated Genes

**Figure 4:**
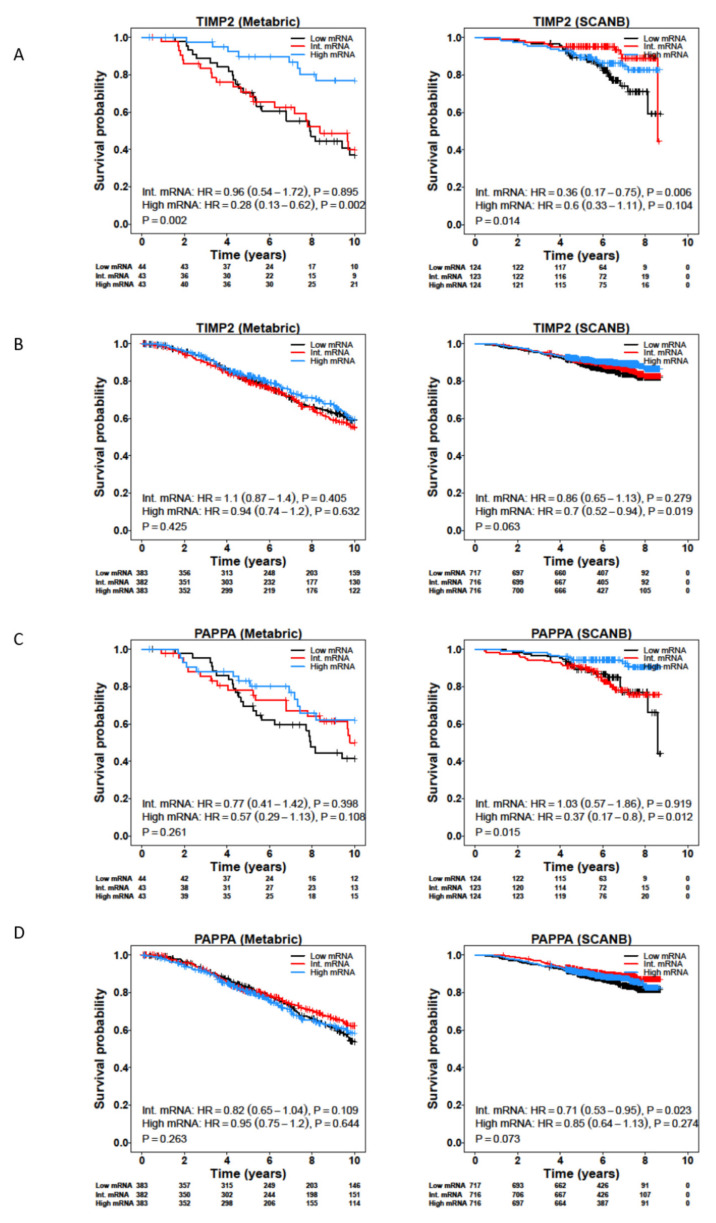
Expression of *TIMP2* and *PAPPA* is associated with better survival in ILC patients. Kaplan–Meier plots for overall survival for *TIMP2* in ER+ ILC (**A**) and IDC (**B**), and *PAPPA* in ER+ ILC (**C**) and IDC (**D**) tumors from the METABRIC and SCAN-B studies. Patients were divided into tertiles based on their *TIMP2* and *PAPPA* mRNA levels.

We carried out survival analysis on the CAF-associated genes identified in both the LCM and CAF datasets, using the METABRIC, TCGA, SCAN-B and Desmedt cohorts (Supplementary Tables S4 and S5). While most of the genes were not associated with survival, *TIMP2* showed a positive association with better survival in ILC, but not IDC, across the METABRIC, SCAN-B and Desmedt cohorts (Figure 4A,B; Supplementary Tables S4 and S5). In addition, low expression of *PAPPA* was associated with worse survival for ILC patients in both the SCAN-B and Desmedt cohorts (Figure 4C,D). *PAPPA* showed the greatest fold-change expression in the stromal compared to epithelial compartments in ILC (log2(FC(TS/TE)) = 2.6, FDR-adjusted *p* = 0.000226) (Table 1). *PAPPA* encodes PAPP-A, a secreted metalloproteinase that cleaves IGFBP-4, releasing bioactive IGF-1 [26]. PAPP-A activity can be inhibited by non-covalent or covalent complex formation with endogenous inhibitors stanniocalcin-1 (STC1) or -2 (STC2), respectively [27,28]. Further analysis of IGF pathway related genes identified that *IGF1*, *IGF1R*, *IGFBP4* and *STC2* were all significantly associated with worse survival in both the SCAN-B and METABRIC cohorts, with greater significance for IDC patients compared to ILC patients (Supplementary Tables S4 and S5).

### 3.5. PAPPA Is Predominantly Expressed in the Stroma of ILC

**Figure 5:**
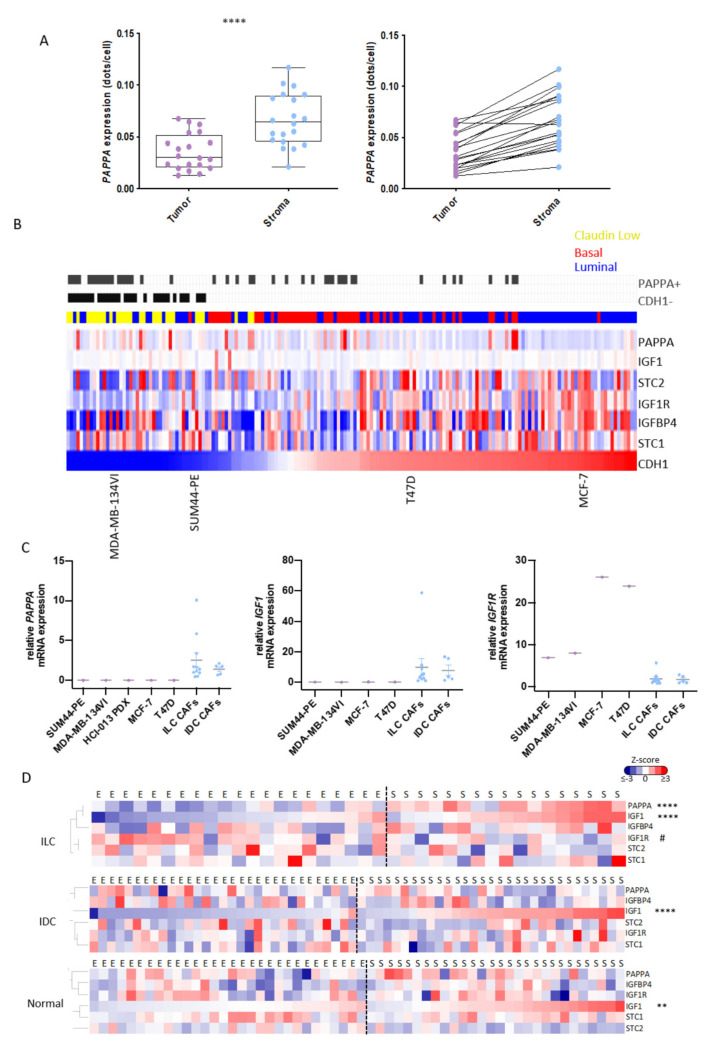
*PAPPA* and *IGF1* are predominantly expressed in the stroma of ILC. (**A**) RNAScope for *PAPPA* was performed in 20 ILC tumor samples that were used for generation of the LCM-ILC dataset. Samples were analyzed using QuPath with transcripts in both the stromal and epithelial compartments quantified. Left: Boxplots representing expression of *PAPPA* (dots per cell) in the tumor and stroma. Paired *t*-test, **** *p* < 0.0001. Right: Trend lines show an increase in *PAPPA* in the stroma of ILC samples compared to the tumor cells. Purple and blue dots represent data from tumor epithelium and stroma, respectively. (**B**) Heatmap representing the expression of *PAPPA* and associated genes across three integrated panels of breast cancer cell lines following batch correction, ranked by *CDH1* expression. Blue, luminal; red, basal; yellow, claudin-low. Grey bars indicate samples where the detection call for *PAPPA* is assigned as ‘present’ and those tumors where *CDH1* is ‘absent’. (**C**) Expression of *PAPPA*, *IGF1* and *IGF1R* by qPCR in ILC human cell lines (purple dots) and primary CAFs (blue dots). Each sample was analyzed at three different passage numbers, and its average represented as the relative mRNA expression to ED30 primary ILC CAFs. Line represents the mean with SEM. (**D**) Heatmaps representing *PAPPA* and its regulators in the epithelial (E) and stromal (S) compartments of ILC-, IDC- (GSE68744) and normal- (GSE4823) LCM datasets. Heatmaps were generated from Z-scored values using Cluster 3.0 and TreeView. Adjusted *p* values were calculated using Wilcox test in R. ** *p* < 0.01, **** *p* < 0.0001, # *p* < 0.05. * Means up in stroma vs. epithelium, # means up in the epithelium vs. stroma.

Expression of PAPP-A in the TE has been reported in a number of different tumor types including breast cancer [29,30], and to verify that *PAPPA* was expressed predominantly by CAFs in ILC, we analyzed *PAPPA* transcripts by RNAScope. Results confirmed higher levels of *PAPPA* expression in fibroblasts compared to tumor cells, although epithelial *PAPPA* transcripts were also seen in some ILC tumors (Figure 5A; Supplementary Figure S5A). Multiplex staining of human ILC tumors confirmed expression of PAPP-A in α-SMA positive fibroblasts with αSMA positive fibroblasts being located throughout the tumors (Supplementary Figure S5B,C). To further explore whether *PAPPA* was a stromal factor, qPCR was carried out in a panel of in vitro models. As there are few cell lines that represent ILC, we first examined *PAPPA* across three integrated breast cancer cell line datasets [31]. *PAPPA* was low or undetectable in all luminal cell lines, including two reported ILC lines: SUM44-PE and MDA-MB-134VI (Figure 5B). qPCR confirmed that *PAPPA* was not expressed in SUM44-PE and MDA-MB-134VI cells or the T47D and MCF-7 ER+ IDC lines, but was in expressed in both ILC and IDC primary patient-derived CAFs (Figure 5C). *IGF1* was also expressed in the CAFs while *IGFR1* was predominantly expressed in the tumor cells: *STC1*, *STC2* and *IGFBP4* were expressed in both tumor cells and CAFs (Figure 5B,C: Supplementary Figure S6A). We also separated tumor epithelial cells from CAFs in tumors derived from a mouse model of ILC driven by loss of *Trp53* and *Cdh1* (Supplementary Figure S7A) [32]. qPCR results showed that *Pappa*, *Igf1* and *Stc1* were only expressed in the CAFs, while *Igf1r*, *Stc2* and *Igfbp4* were expressed in both tumor cells and CAFs (Supplementary Figure S7B).

We then examined expression of *PAPPA* and functionally related genes in the LCM-ILC, -IDC and -normal datasets (Figure 5D). In ILC, both *PAPPA* and *IGF1* were significantly upregulated in the stroma compared to the epithelium (*p* < 0.0001), while *IGF1R* was found predominantly in the tumor epithelium (*p* < 0.05). In IDC and normal breast tissue, *IGF1* was also predominantly expressed in the stroma (*p* < 0.0001 and *p* < 0.01, respectively), whereas *PAPPA* was expressed in both stromal and epithelial compartments (Figure 5D).

**Figure 6:**
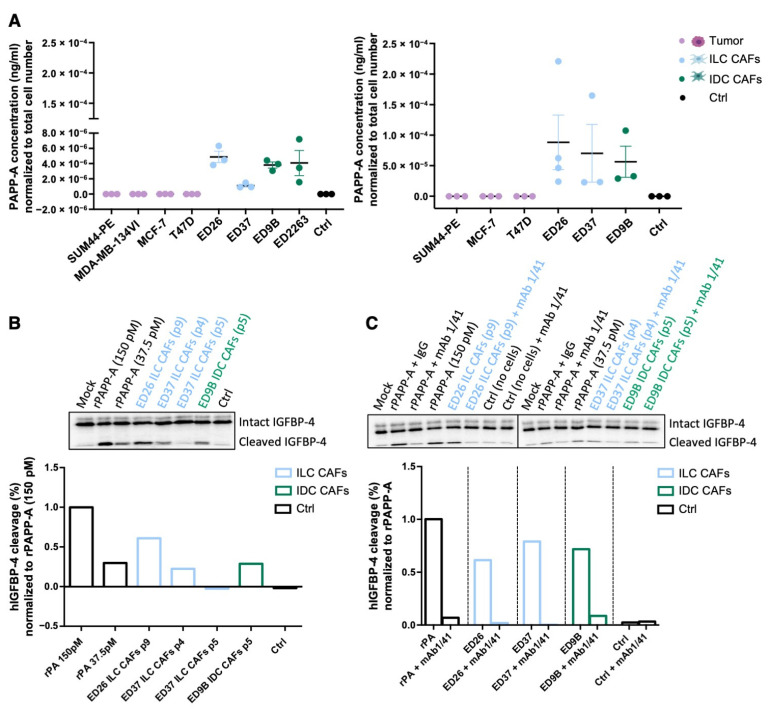
Active PAPP-A is secreted from CAFs. (**A**) ELISA of PAPP-A secreted from a series of ILC and IDC cell lines and primary CAFs. Conditioned medium (CM) was collected after 24 h (left panels) or after 48 h from the tumor cells and 96 h from the CAFs (right panels). PAPP-A concentration was normalized to total cell number (n = 3). (**B**) PAPP-A-mediated IGFBP-4 cleavage. CM from ILC and IDC primary CAFs was incubated with radiolabeled IGFBP-4 for 1, 2 and 4 h. Recombinant PAPP-A (rPAPP-A) was used as a positive control. Quantification at 4 h is represented as the percentage of human IGFBP-4 cleavage normalized to recombinant PAPP-A (150 pM). (**C**) IGFBP-4 cleavage was measured in the presence of PAPP-A inhibitory antibody (mAb 1/41) or isotype control (IgG) in CM from ILC and IDC CAFs. Quantification at 4 h is represented as the percentage of human IGFBP-4 cleavage normalized to the corresponding recombinant PAPP-A (150 pM or 37 pM). *p* denotes passage number of the CAFs.

Analysis of conditioned media (CM) confirmed that PAPP-A was secreted from the patient-derived CAFs but not the tumor cells (Figure 6A). PAPP-A needs to be active in order to cleave IGFBP-4 and liberate IGF-1, and CM from the CAFs was able to cleave recombinant IGFBP-4, indicating that non-complexed, active PAPP-A was present in the media (Figure 6B, the full western blots can be found in Supplementary Material). To confirm that the IGFBP-4 fragments generated by the CAF CM were a result of PAPP-A activity, the CM was treated with a PAPP-A inhibitory antibody (mAb 1/41) [33]. Pre-incubation with mAb 1/41 reduced levels of the IGFBP-4 fragment to those in the control lane, showing that the observed cleavage of IGFBP-4 is due to PAPP-A present in the CM (Figure 6C, the full western blots can be found in Supplementary Material).

Interestingly, we found a clear positive correlation between *PAPPA* and *IGF1* in ILC (r = 0.64, *p* < 0.0001), which was not observed in IDC and normal tissue LCM datasets (Supplementary Figure S6B). A positive and highly significant correlation was also observed between *PAPPA* and both *IGF1* and *IGF1R* expression in bulk ILC ER+ tumors (Supplementary Figure S6C). Increased expression of *IGF1* has been reported previously in ILC compared to IDC [34,35,36], along with reported pathway activation [35,37]. However, *PAPPA* did not significantly correlate with phospho-IGF1R in matched RNA and reverse phase protein array samples from ILC ER+ patients in TCGA dataset (Supplementary Figure S6D).

## 4. Discussion

In this study, we provide the first comprehensive analysis of the TME in ILC. We show that there is a subset of genes involved in ECM interactions and signaling pathways that are enriched in the stroma of ILC compared to IDC, and further analysis of CAFs isolated from ILC and IDC, identified additional differentially expressed genes. There is little known about the role of CAFs in ILC with one study reporting an increased CAF density in ILC compared to matched IDC samples [34], while differences in collagen deposition and alignment have also been reported [38,39]. Taken together with the differential expression of stromal genes involved in ECM organization between ILC and IDC, this indicates that CAFs are able to differentially influence the TME in ILC.

A number of the differentially expressed stromal genes also showed higher expression in bulk ILC tumors compared to IDC. These included genes encoding metalloproteinases (MMPs) and their inhibitors: *ADAMTS16*, *MMP2* and *TIMP2*. ADAMTS16 is a member of the ADAMTS (a disintegrin and metalloprotease with thrombospondin motifs) family of proteases involved in degradation of the ECM. Little is known about its function or regulation, with one study reporting increased expression in IDC compared to DCIS [40], while another study found evidence of reduced protein expression in a number of tumor types due to promoter methylation [41]. MMP2 is an important collagenase which is widely expressed in invasive breast cancers. Interestingly, in a recent study MMP2 was also seen in both lobular carcinoma in situ (LCIS) and paired normal breast tissue [42]. MMP16, a membrane-bound MMP which proteolytically activates MMP2, was also increased in ILC CAFs further supporting an important role for MMP2 activity in ILC. Although there was no correlation between MMP2 and survival in patients with ILC, high levels of *TIMP2* were associated with increased survival. Tissue inhibitor of metalloproteinase 2 (TIMP2) is a matrix metalloproteinase inhibitor with activity against MMP2. However, it is also recognized to have other MMP-independent functions including regulation of tumor angiogenesis, and both increased and decreased expression of TIMP2 has been linked to a more favorable prognosis in breast cancer patients [43]. Importantly, the ratio between MMP2 and TIMP2 appears to be an important factor, and validation of the expression and activity of this pathway in ILC will be important. Furthermore, note that the survival analysis carried out in this study relied on data from bulk tumors, and it will be important in the future to look specifically at the association between stromal expression and survival.

*CECR1*, a secreted adenosine deaminase also known as *ADA2*, was identified as both an ILC stromal gene in the LCMS analysis and as being upregulated in ILC CAFs in the RNA-Seq analysis. *CECR1* has been linked to macrophage polarization to immunosuppressive M2 phenotype in glioma [44,45] and triple-negative breast cancer (TNBC) [46], and ADA2 inhibition in a mouse model of TNBC led to decreased tumor growth in vivo and decreased invasion in vitro [47]. *CECR1* also shows increased expression in aggressive TNBC, although the opposite was found in hormone receptor-positive tumors [46]. Although there was no consistent survival impact of *CECR1* in our analysis, the role of *CECR1* in ILC warrants further investigation.

The pathways enriched in ILC CAFs included mTORC signaling, which has been reported to control the secretion of proinflammatory cytokines from CAFs [48,49]. Previous analysis of bulk ILC tumors identified two distinct subtypes based on gene expression clustering, one of which was termed “immune-related” due to the enrichment of genes involved in immune signaling. Further analysis identified higher levels of lymphocytic infiltration in the “immune-related ILC tumors with specific enrichment of T cell markers [7]. As our study excluded analysis of the immune cell populations it is not possible to correlate expression of the CAF-associated genes with different immune signatures. Understanding the role of CAFs in controlling the unique repertoire of immune cells reported in ER+ ILC [10], and in specific ILC subtypes, will be important. Of note, CAFs in the LCM analysis were identified based on their morphology within the stromal compartment of the tumor, and no marker analysis was carried out to distinguish between different fibroblast populations. A wider analysis of fibroblasts from ILC will be required to fully understand their biological and clinical importance and also whether the heterogeneity and different sub-populations of CAFs that exist in other breast cancers are pertinent to ILC [50,51]. Interestingly, four of the stromal-derived ECM-associated genes (*PRKCA*, *ITGA10*, *NOV*, *WNT5B*) that we identified in ILC were found in the *reactive-like* ILC subtype described by Ciriello and colleagues [5]; these *reactive-like* ILC tumors largely associate with the *reactive* subgroup identified by TCGA that is characterized by strong microenvironmental signaling and CAFs [20].

*PAPPA* was the most enriched gene in the TS compared to TE in ILC. Of the few immunohistochemical studies reporting on PAPP-A in breast tumors, only expression in the tumor epithelium has been recorded [29,30,52]. However, we found that both *PAPPA* and *IGF1* were predominantly expressed in the stroma of ILC, while *IGF1R* was expressed within the tumor epithelium. Together with the demonstration that active PAPP-A is secreted from CAFs and colocalized with α-SMA positive fibroblasts in ILC tumors, this strongly supports the existence of a paracrine signaling pathway in ILC. Due to the lack of specific markers for CAFs, we cannot exclude the possibility that PAPP-A is also secreted from α-SMA myofibroblasts. This could provide an additional paracrine signaling axis in ILC. Further analysis of a panel of breast cancer cell lines showed that *PAPPA* was low or undetectable in all luminal cell lines, suggesting that a PAPP-A paracrine signaling axis is also present in other breast cancer subtypes.

Previously, we have shown that loss of E-cadherin promotes hypersensitization of PI3K/AKT pathway activation in response to IGF1, independent of PAPP-A [35] and of oncogenic mutations in the PI3K/AKT pathway that are prevalent in ILC [5]. This is consistent with other reports demonstrating that E-cadherin-mediated adhesion negatively regulates IGF1R activation [36,37]. Furthermore, in breast cancer models, loss of E-cadherin, and the subsequent activation of IGF1R signaling, results in increased sensitivity to dual IGF1R/Insulin receptor inhibitors, and AKT inhibitors that target downstream receptor pathway activation, even in the presence of activating *PIK3CA* mutations [35,37]. Interestingly, increased expression of *IGF1* is seen in ILC compared to IDC [34,35,53], consistent with reported pathway activation [35,37]. Together, these data suggest that patients with ILC may benefit from treatments targeting the IGF1 signaling pathway.

Although a number of strategies to target the IGF1/IGF1R signaling axis have been tested, results in the clinical setting have been disappointing, with a number of contributory factors including effects on systemic glucose metabolism and associated metabolic toxicities and lack of predictive biomarkers [54]. Indirect targeting of IGF1R signaling via inhibition of PAPP-A proteolytic activity has been proposed as an alternative way to block IGF1R signaling by reducing the levels of bioactive IGF1 specifically in the local microenvironment of tumors that express high levels of PAPP-A [33,55,56,57]. A recent study found that circulating levels of PAPP-A in breast cancer patients were independently prognostic for recurrence-free and overall survival [58]. However, PAPP-A has also been proposed to be a tumor suppressor following the discovery that it is epigenetically silenced in breast cancer precursor lesions [29], and survival analysis in this study, using publicly available breast cancer gene expression datasets, showed that reduced *PAPPA* is associated with worse outcomes in ILC. Although an IGF-1-activated gene signature identified in MCF-7 cells following stimulation with IGF-1 has been correlated with poor prognosis in breast cancer [59], other reports have shown that a gene signature associated with high *IGF1* expression in breast cancer samples is associated with more favorable outcomes, with the authors suggesting that higher levels of *IGF1* drive a more differentiated, less aggressive phenotype in ER+ tumors [60]. The strong association and interplay between IGF-1 and ER signaling adds to the complexity, and it is likely that a combination of markers will be required to identify which patients may benefit from targeting this pathway. It is also important to consider that *PAPPA* levels do not reflect active proteinase activity of the secreted PAPP-A, and indeed *PAPPA* levels did not correlate with levels of activated IGF-1R. PAPP-A is predominantly found bound to its endogenous inhibitors STC1 and STC2, and it will be important in the future to consider measurements of active PAPP-A.

## 5. Conclusions

This study provides the first in-depth characterization of the stromal make-up of ILC, identifying a number of differences in the CAF-associated gene expression profiles between ILC and IDC and highlighting potentially clinically relevant pathways.

## Figures and Tables

**Table 1:** Relative expression of CAF genes enriched in the stroma of ILC in ER+ ILC and IDC tumors. Log_2_(fold changes) indicate log_2_(TS/TE) gene expression changes in the LCM-ILC dataset. All genes in LCM-ILC dataset were significantly enriched in TS vs. TE by rank products (pfp < 0.01). Significance levels shown for individual gene expression levels in METABRIC and TCGA bulk tumor datasets. ILC or IDC indicates significant increase in gene expression in that subtype; Mann–Whitney U test, * *p* < 0.05, *** *p* < 0.0001, ns = no significance difference between ILC and IDC.

Genes	Log2 fold Change (TS/TE)	METABRIC	TCGA Microarray	TCGA RNA-Seq
*PAPPA*	2.59	ILC (*)	ILC (*)	ILC (*)
*MMP2*	2.29	ns	ILC (*)	ILC (***)
*ANGPTL2*	2.02	ILC (*)	ns	ILC (***)
*TIMP2*	1.92	ns	ns	ILC (*)
*NID1*	1.89	ns	ns	ns
*NOV*	1.85	ns	ILC (***)	ILC (***)
*EFEMP2*	1.71	IDC (***)	ns	ILC (***)
*CAPN5*	1.61	ILC (*)	ns	ILC (***)
*PRKCA*	1.57	ILC (*)	ILC (*)	ILC (***)
*COL18A1*	1.41	IDC (*)	ns	ILC (***)
*SERPINH1*	1.25	IDC (*)	ns	ns
*TNC*	1.07	ns	ILC (*)	ILC (*)
*MMP14*	1.05	ns	ns	ns
*COL6A3*	1.04	ns	ns	ns
*COLEC11*	1.00	ILC (*)	ns	ns
*CD109*	0.99	IDC (*)	ns	ns
*TNFSF9*	0.94	IDC (*)	ns	ns
*LOX*	0.90	ns	ns	ns
*ITGA7*	0.87	ILC (*)	ILC (***)	ILC (***)
*GPC6*	0.83	IDC (*)	ns	IDC (***)
*HAPLN3*	0.81	ns	ns	ILC (***)
*ITGA10*	0.81	ILC (***)	ILC (***)	ILC (***)
*BMPER*	0.72	ns	ns	ILC (***)
*GDF11*	0.67	IDC (***)	ns	ns
*TGFB3*	0.65	ILC (***)	ILC (*)	ILC (***)
*WNT5B*	0.63	ns	ns	ILC (*)
*BMP2*	0.61	ns	ILC (*)	ILC (***)
*CAPN3*	0.59	ns	ns	ILC (***)

**Table 2:** Genes encoding secreted proteins are significantly upregulated in ILC CAFs. Expression of the 8 significant (Mann–Whitney U test, *p* < 0.05) secreted protein-encoding genes upregulated in ILC CAFs compared to IDC CAFs in CAF RNA-Seq dataset. Log_2_(fold changes) for CAF RNA-Seq dataset are log_2_(ILC/IDC) with corresponding *p*-values. Significance levels shown for individual gene expression levels in METABRIC and TCGA bulk tumor datasets. ILC or IDC indicates significant increase in expression in that subtype; Mann–Whitney U test, * *p* < 0.05, *** *p* < 0.0001, ns = not significant; NA = data not available.

Genes	Log2 Fold Change (ILC/IDC)	*p*-Value	METABRIC	TCGA Microarray	TCGA RNA-Seq
*CECR1*	1.36	0.042	ns	ILC (*)	ILC (*)
*MMP16*	1.66	0.024	ns	ns	ns
*IL1A*	2.20	0.014	ns	ns	ns
*NPW*	2.82	0.007	NA	ILC (*)	ns
*ADAMTS16*	3.07	0.044	ns	ILC (*)	ILC (*)
*GATD3A*	4.49	0.027	ns	ns	ns
*CHGA*	4.69	0.028	ns	ns	IDC (***)
*MTRNR2L8*	4.08	0.003	NA	NA	NA

## Data Availability

Processed and raw data are available from Gene Expression Omnibus (GSE148398, GSE148156).

## Supplementary Materials

The following supporting information can be downloaded at: https://www.mdpi.com/article/10.3390/cancers14040904/s1. Figure S1: Overview of laser-capture microdissection technique. Figure S2: Expression of stromal genes enriched in ILC analyzed in METABRIC, TCGA RNA-Seq and TCGA microarray datasets. Figure S3: Characterization of primary CAFs. Figure S4: ILC stromal genes identified by LCMS expressed in ILC and IDC CAFs. Figure S5. PAPPA mRNA and PAPP-A protein expression in ILC. Figure S6: Expression of PAPPA and IGF1 associated genes in cell lines and tumor datasets. Figure S7: Pappa and Igf1 associated gene expression in ILC mouse model. Table S1: Pathway enrichment analysis of stromal genes enriched in ILC. Table S2. Expression of the 17 ILC-stromal ECM genes not expressed in CAFs in IDC and ILC ER+ tumours. Table S3: Significantly differentially expressed ILC stromal genes between primary ILC and IDC CAFs. Table S4 and S5: Survival analysis of ILC stroma and CAF-associated genes in ILC and IDC patients. Table S6: Primers used for RT-qPCR analysis. Supplementary Methods and the full western blots of Figure 6B,C. [14,23,61,62,63,64].
