# Supplementary material for: Characterisation of the Stromal Microenvironment in Lobular Breast Cancer
Source: Cancers (Basel). 2022 Feb 11;14(4):904. doi: 10.3390/cancers14040904 (PMC8870100; doi:10.3390/cancers14040904)
Supplement: Supplementary file 1 [file cancers-14-00904-s001.zip › cancers-1544948-supplementary.pdf]

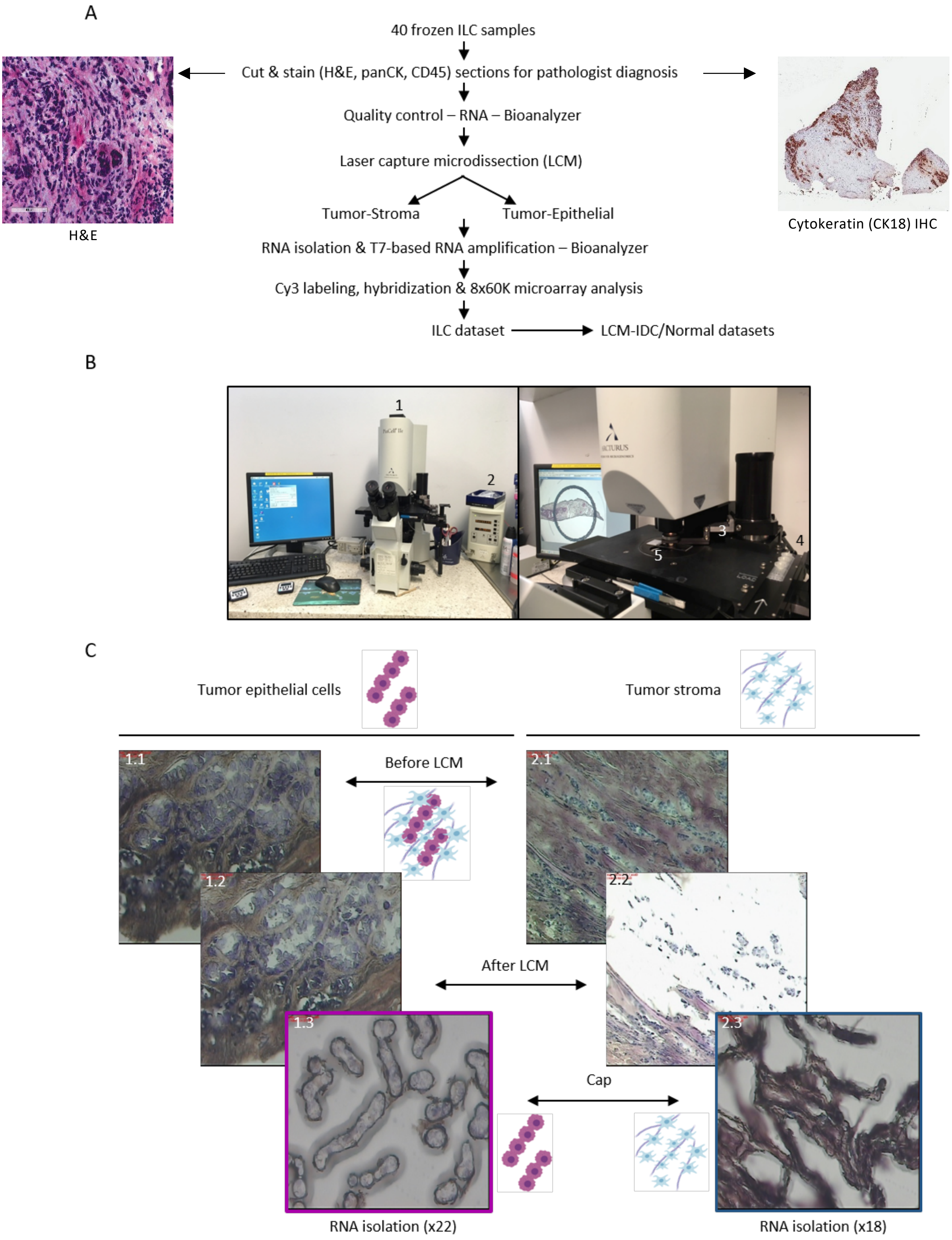

Figure S1: Overview of laser-capture microdissection technique. Workflow used for the generation of a lobular gene expression dataset comprised of ILC epithelial (TE) and stromal cells (TS). B) PixCell IIe Laser Capture Microdissection microscope (1). An infrared laser (2) is used to lift the cells of interest. A handle (3) is used to place the HS Capture cap (4) on the top of the HistoBond stained slide (5). C) Images of two ILC tissue samples being processed. Left: isolation of tumor epithelial cells. Right: isolation of tumor stromal cells. 1.1 & 2.1) Tissue before LCM; 1.2 & 2.2) Tissue after LCM; 1.3 & 2.3) Caps after LCM. A total of 22 and 18 epithelial and stromal samples were isolated respectively from 23 ILC samples, 17 of them paired-matched.

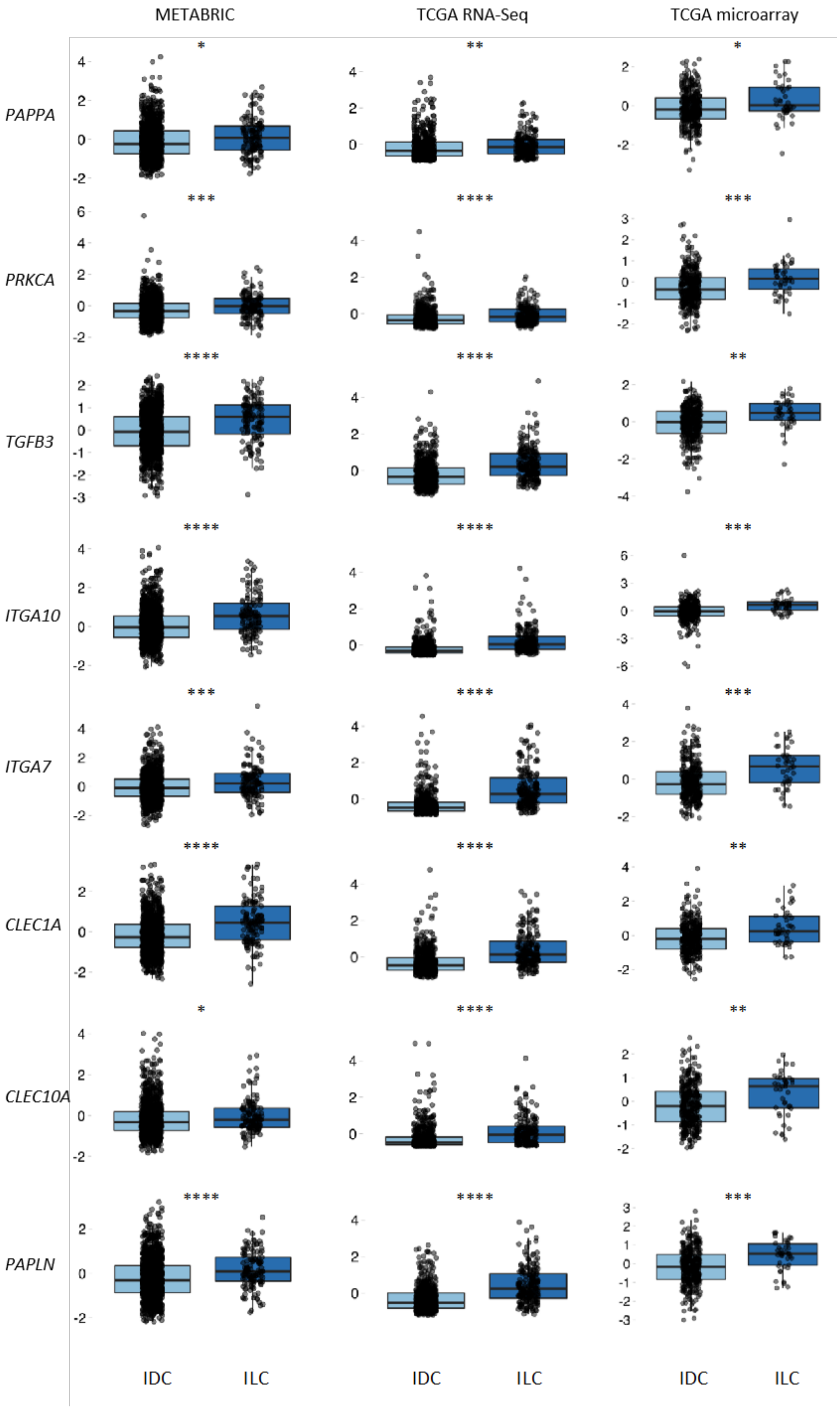

Figure S2: Expression of stromal genes enriched in ILC analyzed in METABRIC, TCGA RNA-Seq and TCGA microarray datasets. Boxplots of expression for TS-ILC enriched genes from METABRIC (Illumina v3 microarray) and TCGA provisional (RNA-Seq v2 and TCGA microarray) mRNA expression datasets (expression Z-scores; Z-score threshold ±2). Analysis was reduced to ILC and IDC ER-positive samples only. Boxplots display the median (line), 25th and 75th percentiles (box) and interquartile range (whiskers). Light blue, IDC; dark blue, ILC. *p ≤ 0.05, **p ≤ 0.01, ***p ≤ 0.001, ****p ≤ 0.0001; Wilcoxon test.

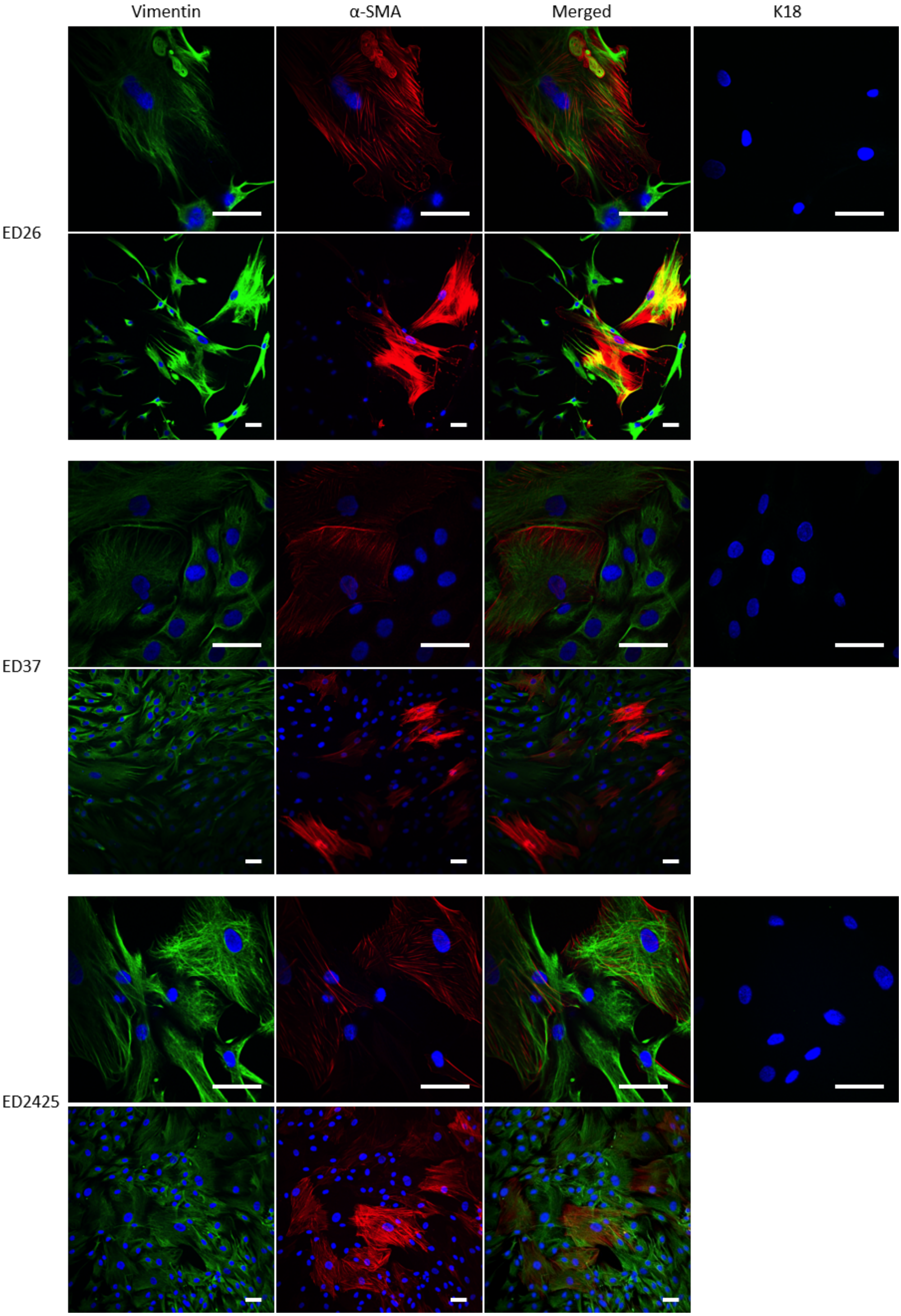

Figure S3: Characterization of primary CAFs. Primary CAFs stained for the mesenchymal markers vimentin and α-SMA, and the epithelial marker keratin18. Examples provided for three ILC derived CAF lines. Scale bar, 50 µm.

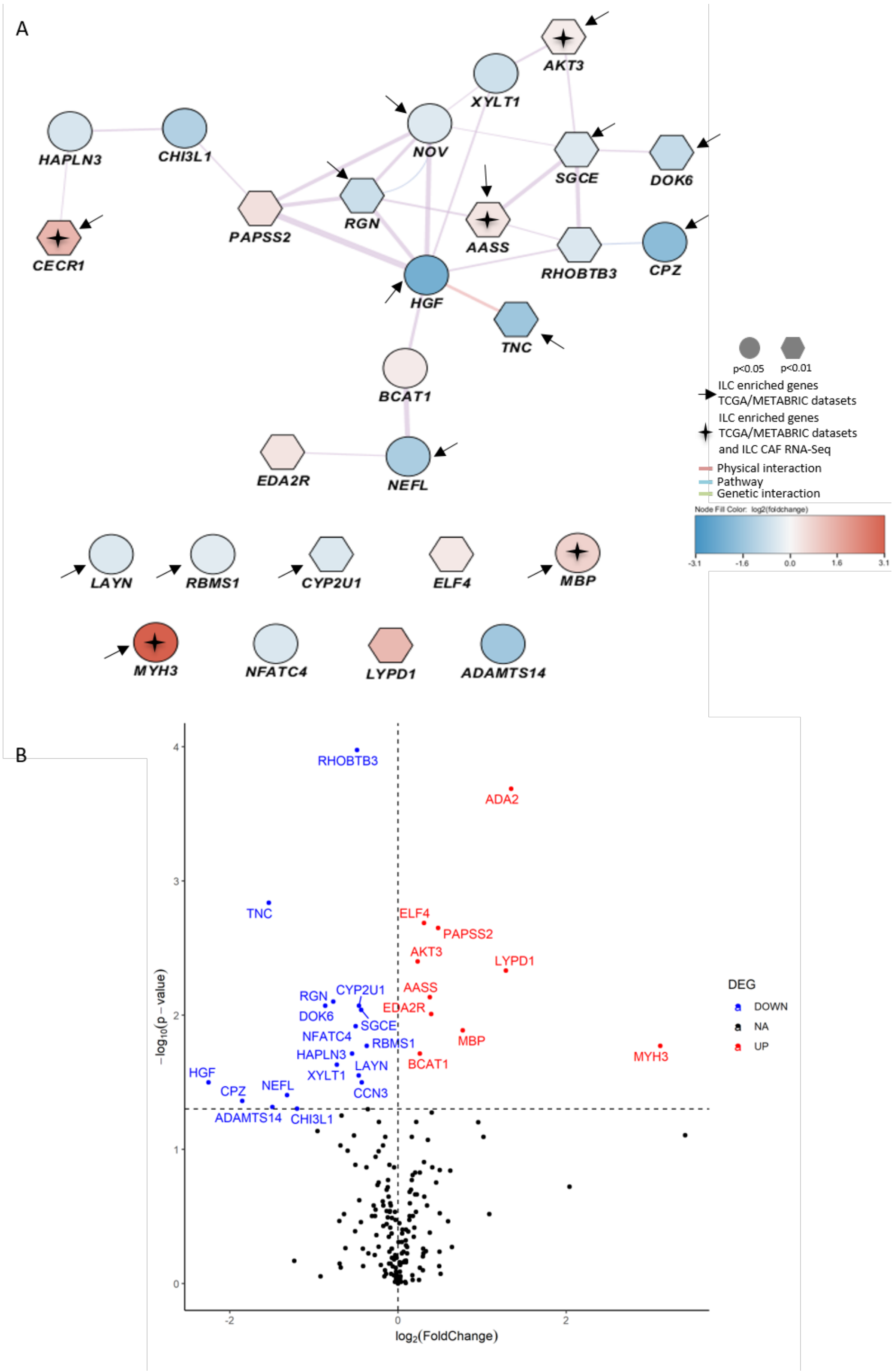

Figure S4: ILC stromal genes identified by LCMS expressed in ILC and IDC CAFs. A) GeneMANIA network created in Cytoscape showing the 27 ILC stromal genes significantly differentially expressed between ILC and IDC CAFs in the RNA-Seq dataset. Positive log fold change shows higher expression in ILC CAFs (in red) and negative log fold change shows higher expression in IDC CAFs (blue). Significance was determined by Mann-Whitney U test. Hexagons represent $p<0.01$, circles $p<0.05$, arrows indicate genes upregulated in ILC tumours compared to IDC tumours in TCGA and METABRIC datasets, with crosses indicating genes upregulated in both ILC CAFs and tumours. Network connectors represent co-expression (purple), physical interactions (pink), and co-localisation (blue). B) Volcano plot of ILC stromal genes determined by LCM analysis expressed in ILC and IDC CAFs. Genes were considered differentially expressed if $p<0.05$ (Mann-Whitney test) with genes more highly expressed in ILC CAFs labelled in red and in IDC CAFs in blue.

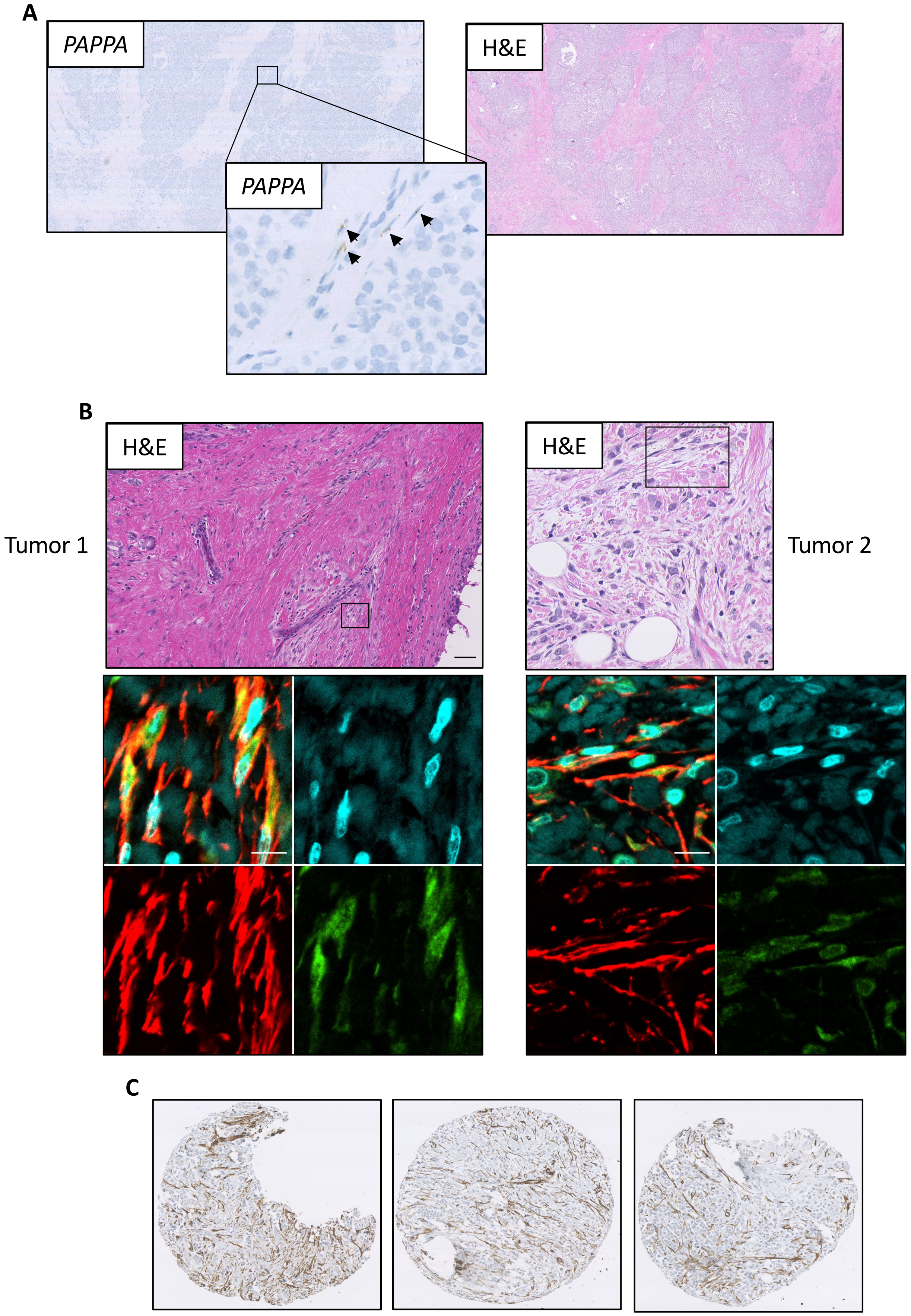

Figure S5. *PAPPA* mRNA and PAPP-A protein expression in ILC. A) Representative image of RNAScope analysis of *PAPPA* in human ILC with corresponding H&E stained samples. Upper panels, magnification x5; lower panel inset, magnification x40. Arrows show PAPPA transcripts in fibroblasts. B) Multiplex immunocytochemistry was carried out on paraffin embedded human ILC tumors using antibodies to PAPP-A (green) and α-SMA (red) with NucBlue nuclear stain (cyan). Upper panels corresponding H&E stained samples shown for orientation at different magnifications. Scale bar 50 μm (tumor 1); 10 μm (tumor 2). Lower panels are higher magnification IF images showing co-localization of PAPP-A in αSMA-positive CAFs. Representative examples from 2 different tumors shown. Images were collected on an Olympus FV1000 confocal microscope. Scale bar 10 μm. C) Representative images of αSMA IHC in ILC.

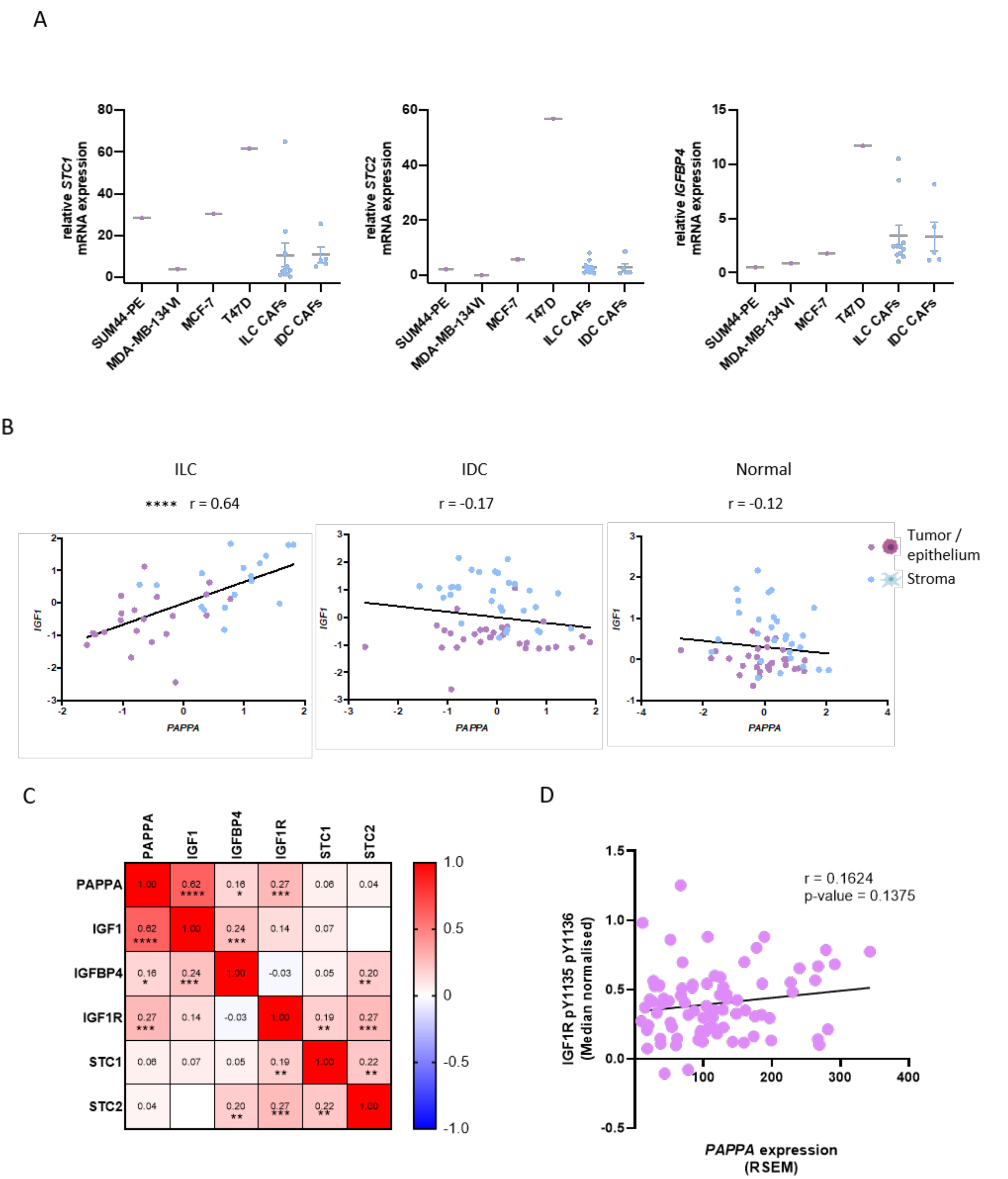

Figure S6: Expression of *PAPPA* and *IGF1* associated genes in cell lines and tumor datasets. A) Expression of *STC1, SCT2* and *IGFBP4* by qPCR in ILC human cell lines and primary CAFs. Each sample was analyzed at three different passage numbers, and its average represented as the relative mRNA expression to ED30 primary ILC CAFs. Line represents the mean with SEM. B) Scatterplots representing *PAPPA–IGF1* correlation in ILC-, IDC- (GSE68744) and normal- (GSE4823) LCM datasets. Pearson correlation and linear regression analyses were performed in GraphPad. ****$p \leq 0.0001$. For (A) and (B), purple dots represent data from tumour epithelium and epithelial cells and blue dots represent data from tumour stroma and stromal cells. C) Correlation matrix of *PAPPA* and *IGF1* related genes expression in ILC ER+ patients, n=191, TCGA BRCA RNA-Seq V2 dataset. Pearson correlation r value plotted, with * indicating p-value (* $p<0.05$, ** $p<0.01$, *** $p<0.001$, **** $p<0.0001$). D) Scatterplot representing *PAPPA* expression (TCGA BRCA RNA-Seq V2) vs pIGF1R protein (TCGA RPPA L4) correlation in ILC ER+ patients (n=85). Pearson correlation and linear regression analyses were performed in GraphPad.

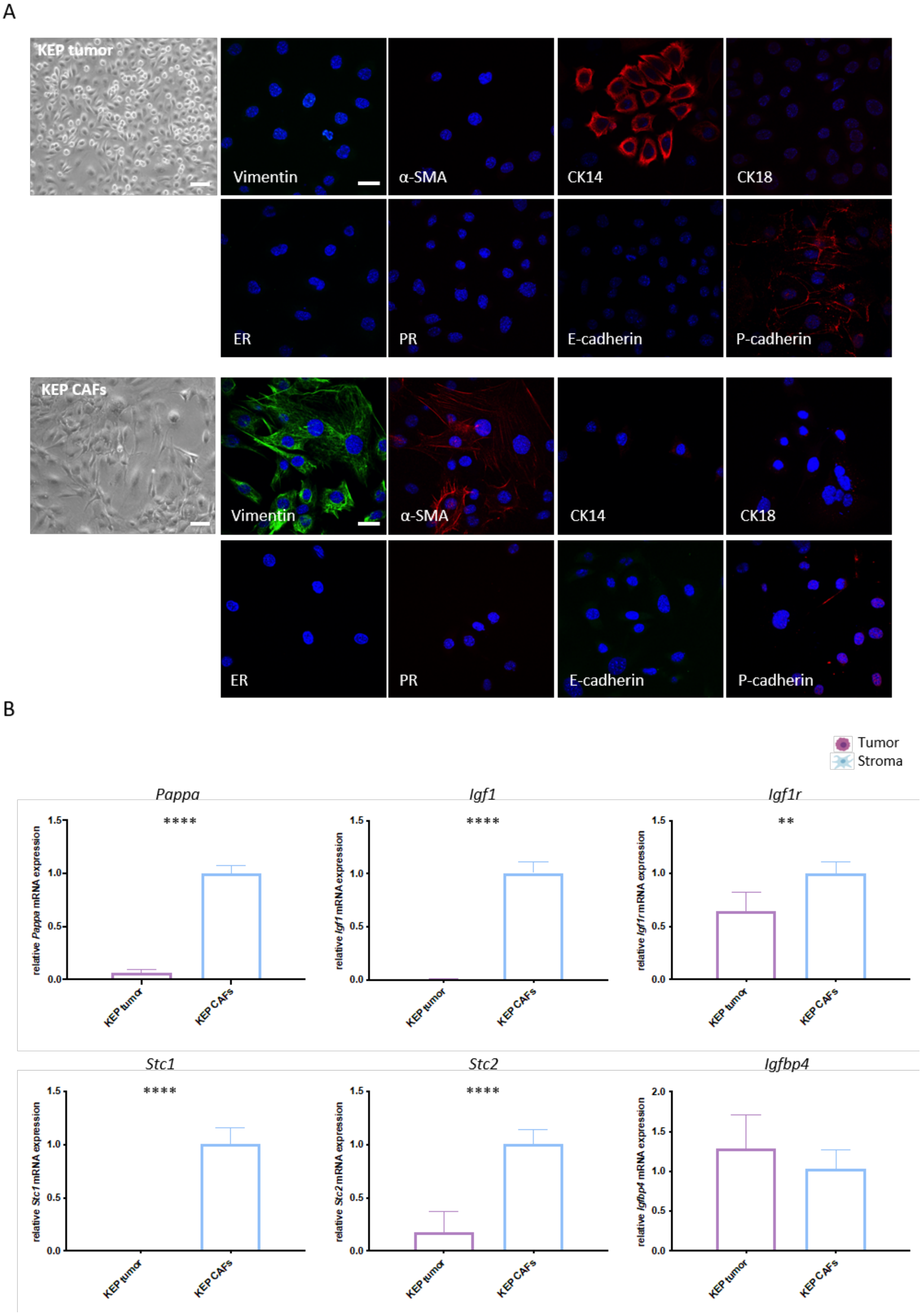

Figure S7: *Pappa* and *Igf1* associated gene expression in ILC mouse model. A) Characterization of tumor and CAF cells derived from the KEP mouse model of ILC by immunofluorescence. B) Expression of *Pappa, Igf1, Igf1r, Sct1, Sct2* and *Igfbp4* by qPCR in KEP tumour cells and CAFs. Each sample was analyzed at three different passages, and its average represented as the relative mRNA expression to KEP CAFs. Values are mean with SEM.

Supplementary Tables

	Pathway	Source	q-value FDR B&H	45 genes merged
Toppgene	Ensemble of genes encoding extracellular matrix and extracellular matrix-associated proteins	MSigDB C2 BIOCARTA (v6.0)	7.13E-05	*ADAMTS14, ADAMTS18, ANGPT2, ANGPTL2, ASTL, BMP2, BMPER, CAPN3, CAPN5, CD109, CILP2, CLEC10A, CLEC1A, COL18A1, COL6A3, COLEC11, DHH, EFEMP2, FAM20A, FCN1, GDF11, GPC6, HAPLN3, HGF, ITGA10, ITGA7, LOX, MMP14, MMP2, MUC19, NID1, NOV, PAPLN, PAPPA, PGF, PRKCA, SERPINH1, SPOCK2, TDGF1, TGFB3, TIMP2, TNC, TNFSF9, WNT5B, ZP1*
	Extracellular matrix organization	BioSystems: REACTOME	1.32E-04	
	Ensemble of genes encoding ECM-associated proteins including ECM-affilaited proteins, ECM regulators and secreted factors	MSigDB C2 BIOCARTA (v6.0)	6.98E-03	

Table S1: Pathway enrichment analysis of stromal genes enriched in ILC. ToppGene pathway enrichment analysis of 261 genes (Benjamini-Hochberg adjusted p-value < 0.05) identified 45 genes involved in significant pathways related to the ECM.

Genes	Log_2 fold change (TS/TE)	METABRIC	TCGA microarray	TCGA RNA-Seq
ADAMTS14	1.83	IDC (*)	ns	ns
PGF	1.71	ns	ns	ILC (***)
CILP2	1.67	ns	ns	ILC (***)
CLEC1A	1.61	ILC (***)	ILC (*)	ILC (***)
FAM20A	1.22	ns	ILC (*)	ILC (***)
HGF	1.08	ns	ILC (*)	ILC (***)
ANGPT2	0.95	IDC (*)	ns	IDC (*)
SPOCK2	0.86	ns	ILC (*)	ILC (***)
PAPLN	0.85	ILC (***)	ILC (*)	ILC (***)
ZP1	0.83	ns	ns	ns
FCN1	0.78	ns	ns	ILC (***)
ASTL	0.73	ns	IDC (*)	ILC (*)
DHH	0.72	ns	ns	ILC (***)
CLEC10A	0.65	ILC (*)	ILC (*)	ILC (***)
ADAMTS18	0.62	ns	ns	ILC (***)
TDGF1	0.61	ns	ns	ILC (***)
MUC19	0.45	ns	ns	ns

Table S2. Expression of the 17 ILC-stromal ECM genes not expressed in CAFs in IDC and ILC ER+ tumours. Log_2(fold changes) indicate log_2(TS/TE) gene expression changes in the LCM-ILC dataset. All genes in LCM-ILC dataset were significantly enriched in TS vs TE by rank products (pfp < 0.01). Significance levels shown for individual gene expression levels in METABRIC and TCGA bulk tumor datasets. ILC or IDC indicates significant increase in expression in that subtype; Mann–Whitney U test, *p < 0.05, ***p < 0.0001, ns = no significance difference between ILC and IDC.

Genes	Log_2 (ILC/IDC)	p-value	Subtype	METABRIC	TCGA microarray	TCGA RNA-Seq
RHOBTB3	-0.49	0.000	IDC	ns	ns	IDC (***)
CECR1	1.35	0.000	ILC	ns	ILC (*)	ILC (*)
TNC	-1.54	0.001	IDC	ns	ILC (*)	ILC (*)
ELF4	0.31	0.002	ILC	ns	ns	IDC (*)
PAPSS2	0.48	0.002	ILC	NA	ns	ns
AKT3	0.23	0.004	ILC	ns	ILC (**)	ILC (***)
LYPD1	1.28	0.005	ILC	IDC (**)	ns	ns
AASS	0.38	0.007	ILC	ILC (**)	ILC (***)	ILC (***)
RGN	-0.77	0.008	IDC	ILC (*)	ILC (**)	ILC (***)
CYP2U1	-0.46	0.009	IDC	ILC (**)	ILC (*)	ILC (**)
DOK6	-0.86	0.009	IDC	ILC (***)	ILC (**)	ILC (***)
SGCE	-0.44	0.009	IDC	ILC (**)	ILC (**)	ILC (***)
EDA2R	0.39	0.010	ILC	ns	ns	ILC (**)
NFATC4	-0.50	0.012	IDC	NA	ns	ILC (**)
MBP	0.77	0.013	ILC	ILC (*)	ILC (*)	ILC (**)
RBMS1	-0.37	0.017	IDC	ns	ILC (**)	ILC (***)
MYH3	3.12	0.017	ILC	ns	ILC (*)	ILC (***)
HAPLN3	-0.55	0.019	IDC	ns	ns	ILC (***)
BCAT1	0.26	0.019	ILC	IDC (***)	ns	IDC (**)
XYLT1	-0.73	0.023	IDC	IDC (*)	ns	ns
LAYN	-0.47	0.028	IDC	ILC (*)	ILC (*)	ILC (***)
HGF	-2.25	0.032	IDC	ns	ILC (**)	ILC (***)
NOV	-0.43	0.032	IDC	ns	ILC (***)	ILC (***)
NEFL	-1.32	0.039	IDC	ns	ILC (***)	ILC (***)
CPZ	-1.85	0.044	IDC	ILC (**)	ILC (*)	ILC (***)
ADAMTS14	-1.49	0.048	IDC	IDC (**)	ns	ns
CHI3L1	-1.20	0.050	IDC	NA	ns	ILC (***)

Table S3: Significantly differentially expressed ILC stromal genes between primary ILC and IDC CAFs. Log_2(fold changes) for CAF RNA-Seq dataset are log_2(ILC/IDC) with corresponding p-values and CAF subtype in which gene is upregulated indicated. Significance levels shown for individual gene expression levels in METABRIC and TCGA bulk tumor datasets. ILC or IDC indicates significant increase in expression in that subtype; Mann–Whitney U test, *p < 0.05, **p < 0.01 ***p < 0.0001, ns = no significance difference between ILC and IDC; NA = data not available.

		ILC											
		TCGA BRCA (n=203)			METABRIC (n=147)			SCANB (n=386)			Desmedt (n=117)		
**Gene**	**Identified by**	HR	CI	p-value	HR	CI	p-value	HR	CI	p-value	HR	CI	p-value
ANGPTL2	LCMS	0.41	0.14-1.2	0.224	0.48	0.25-0.91	0.071	0.54	0.3-0.97	0.012	0.54	0.16-1.8	0.487
CAPN5	LCMS	0.55	0.21-1.46	0.153	0.6	0.33-1.11	0.195	0.68	0.37-1.26	0.446	0.78	0.3-2.04	0.136
ITGA10	LCMS	0.32	0.12-0.91	0.008	0.64	0.35-1.19	0.359	0.86	0.48-1.56	0.450	1.26	0.42-3.76	0.854
ITGA7	LCMS	0.66	0.23-1.91	0.740	0.93	0.52-1.68	0.862	0.54	0.29-1.01	0.068	0.61	0.2-1.87	0.672
MMP2	LCMS	0.67	0.25-1.82	0.342	0.65	0.33-1.26	0.024	0.72	0.4-1.31	0.290	0.98	0.33-2.9	0.998
PRKCA	LCMS	0.9	0.31-2.58	0.979	0.53	0.29-0.97	0.053	0.68	0.37-1.23	0.136	1	0.38-2.59	0.142
TGFB3	LCMS	0.61	0.21-1.81	0.640	0.54	0.3-0.99	0.095	0.68	0.36-1.26	0.465	0.21	0.04-0.96	0.057
TIMP2	LCMS	0.62	0.2-1.86	0.665	0.31	0.15-0.63	0.002	0.56	0.31-1	0.014	0.53	0.13-2.1	0.049
IGF1	IGF1 pathway	0.57	0.21-1.59	0.476	0.67	0.37-1.22	0.417	0.44	0.22-0.85	0.039	0.44	0.15-1.28	0.140
IGF1R	IGF1 pathway	0.53	0.18-1.49	0.452	0.81	0.45-1.45	0.314	0.41	0.22-0.76	0.003	0.97	0.36-2.58	0.363
IGFBP4	IGF1 pathway	0.46	0.16-1.34	0.216	0.47	0.25-0.91	0.069	0.4	0.21-0.79	0.023	0.84	0.22-3.11	0.066
PAPPA	LCMS/IGF1 pathway	0.98	0.37-2.62	0.681	0.66	0.36-1.22	0.365	0.38	0.18-0.8	0.005	0.09	0.01-0.71	0.019
STC1	IGF1 pathway	0.53	0.19-1.49	0.382	1.42	0.78-2.6	0.454	1.04	0.57-1.89	0.542	0.48	0.14-1.6	0.393
STC2	IGF1 pathway	0.44	0.17-1.15	0.005	0.52	0.28-0.95	0.060	0.34	0.17-0.67	0.002	0.77	0.23-2.52	0.574
AASS	LCMS + CAF	0.27	0.09-0.84	0.013	0.71	0.38-1.32	0.548	0.51	0.27-0.98	0.124	0.4	0.12-1.26	0.253
AKT3	LCMS + CAF	1.3	0.46-3.66	0.873	0.61	0.33-1.15	0.295	0.46	0.23-0.91	0.057	0.91	0.28-2.97	0.490
EDA2R	LCMS + CAF	0.68	0.23-2.02	0.702	0.85	0.46-1.59	0.680	0.71	0.38-1.31	0.405	1.48	0.42-5.24	0.229
MBP	LCMS + CAF	0.75	0.28-2.02	0.060	0.73	0.4-1.32	0.195	NA	NA	NA	0.88	0.31-2.53	0.881
MYH3	LCMS + CAF	0.56	0.19-1.66	0.541	0.93	0.49-1.74	0.511	0.65	0.33-1.27	0.206	0.75	0.26-2.17	0.788
ADAMTS16	CAF secretome	0.86	0.31-2.39	0.385	0.77	0.41-1.48	0.041	0.84	0.46-1.51	0.590	1.16	0.37-3.59	0.782
C21orf33	CAF secretome	1.54	0.56-4.24	0.360	0.55	0.29-1.04	0.151	1.15	0.62-2.13	0.778	2.51	0.77-8.18	0.288
CECR1	LCMS + CAF secretome	0.7	0.26-1.89	0.412	0.86	0.47-1.59	0.746	1.17	0.66-2.07	0.043	0.64	0.23-1.8	0.417
CHGA	CAF secretome	0.63	0.22-1.82	0.646	0.42	0.23-0.76	0.000	0.56	0.29-1.09	0.216	0.97	0.31-3	0.598
IL1A	CAF secretome	0.61	0.22-1.69	0.450	0.74	0.39-1.39	0.384	0.87	0.48-1.59	0.518	1.27	0.39-4.17	0.481
MMP16	CAF secretome	0.78	0.28-2.17	0.444	0.71	0.39-1.3	0.504	0.46	0.24-0.88	0.049	0.96	0.34-2.74	0.897
MTRNR2L8	CAF secretome	NA	NA	NA	NA	NA	NA	1.16	0.63-2.12	0.694	NA	NA	NA
NPW	CAF secretome	0.31	0.08-1.13	0.155	NA	NA	NA	1.27	0.69-2.32	0.665	1.17	0.42-3.23	0.690

		IDC								
		TCGA BRCA (n=782)			METABRIC (n=1543)			SCANB (n=2598)		
**Gene**	**Identified by**	HR	CI	p-value	HR	CI	p-value	HR	CI	p-value
ANGPTL2	LCMS	1.41	0.86-2.3	0.386	1.13	0.93-1.39	0.419	0.91	0.71-1.16	0.442
CAPN5	LCMS	0.72	0.43-1.22	0.242	1.12	0.91-1.36	0.532	0.7	0.55-0.9	0.007
ITGA10	LCMS	0.71	0.43-1.16	0.249	0.64	0.52-0.78	0.000	0.56	0.43-0.72	0.000
ITGA7	LCMS	0.78	0.47-1.27	0.544	0.94	0.78-1.15	0.373	0.61	0.48-0.78	0.000
MMP2	LCMS	1.07	0.67-1.72	0.958	0.96	0.78-1.17	0.735	0.87	0.68-1.12	0.534
PRKCA	LCMS	1.09	0.68-1.75	0.888	1.03	0.84-1.25	0.709	0.77	0.6-0.99	0.072
TGFB3	LCMS	0.95	0.58-1.55	0.627	0.67	0.55-0.82	0.000	0.44	0.34-0.57	0.000
TIMP2	LCMS	1.39	0.83-2.32	0.206	1.01	0.82-1.23	0.513	0.78	0.61-1.01	0.152
IGF1	IGF1 pathway	0.7	0.44-1.13	0.230	0.85	0.7-1.04	0.265	0.52	0.4-0.66	0.000
IGF1R	IGF1 pathway	0.5	0.3-0.83	0.011	0.56	0.46-0.68	0.000	0.48	0.37-0.61	0.000
IGFBP4	IGF1 pathway	0.54	0.33-0.87	0.017	0.49	0.4-0.61	0.000	0.5	0.38-0.64	0.000
PAPPA	LCMS/IGF1 pathway	0.89	0.53-1.48	0.718	1.03	0.85-1.26	0.665	0.97	0.79-1.23	0.040
STC1	IGF1 pathway	1.28	0.79-2.06	0.534	1.18	0.96-1.46	0.000	1.14	0.88-1.49	0.083
STC2	IGF1 pathway	0.65	0.4-1.07	0.237	0.56	0.45-0.69	0.000	0.47	0.37-0.61	0.000
AASS	LCMS + CAF	0.83	0.51-1.34	0.740	0.88	0.72-1.07	0.440	0.52	0.4-0.67	0.000
AKT3	LCMS + CAF	0.87	0.53-1.43	0.281	1.11	0.91-1.35	0.526	0.67	0.52-0.86	0.004
EDA2R	LCMS + CAF	1.73	1.05-2.84	0.070	0.96	0.79-1.17	0.858	0.6	0.47-0.77	0.000
MBP	LCMS + CAF	0.82	0.5-1.36	0.280	1.03	0.85-1.26	0.944	NA	NA	NA
MYH3	LCMS + CAF	0.65	0.4-1.05	0.182	0.95	0.78-1.15	0.856	0.69	0.53-0.89	0.014
ADAMTS16	CAF secretome	1.19	0.73-1.94	0.773	0.89	0.73-1.08	0.500	0.86	0.67-1.1	0.455
C21orf33	CAF secretome	0.85	0.53-1.36	0.530	0.94	0.77-1.15	0.658	0.8	0.62-1.03	0.205
CECR1	LCMS + CAF secretome	0.47	0.28-0.79	0.014	0.85	0.7-1.04	0.096	0.96	0.74-1.25	0.117
CHGA	CAF secretome	0.6	0.37-0.99	0.130	0.83	0.68-1.01	0.167	0.84	0.66-1.08	0.194
IL1A	CAF secretome	0.62	0.37-1.02	0.163	1.15	0.94-1.39	0.189	1.1	0.85-1.41	0.760
MMP16	CAF secretome	1	0.62-1.62	1.000	1.21	0.99-1.47	0.123	0.46	0.35-0.61	0.000
MTRNR2L8	CAF secretome	NA	NA	NA	NA	NA	NA	1.12	0.88-1.43	0.108
NPW	CAF secretome	1.11	0.68-1.83	0.903	NA	NA	NA	1.32	1.03-1.7	0.068

Table S4 and S5: Survival analysis of ILC stroma and CAF-associated genes in ILC (Table 3) and IDC (Table 4) patients. HR=hazard ratio of high vs low expression groups, CI = 95% confidence interval for HR of high and low expression groups, LCMS = genes identified as ILC stromal genes by laser capture microdissection analysis, CAF secretome = identified by analysis of genes encoding secreted proteins upregulated in ILC CAFs, LCMS + CAFs = genes identified by LCMS analysis and upregulated in ILC CAFs vs IDC CAFs. NA = gene not available in this study.

Primers	Forward 5' - 3'	Reverse 5' - 3'	Species	Tm
hGAPDH	GGACCTGACCTGCCGTCTAG	TGGTGCTCAGTGTAGCCCAG	human	Tm = 56-62 ^{o}C
hIGF1	CTTCAGTTCGTGTGTGGAGACAG	CGCCCTCCGACTGCTG	human	Tm = 59 ^{o}C
hIGF1R	CTCCTGTTTCTCTCCGCCG	ATAGTCGTTGCGGATGTCGAT	human	Tm = 57 ^{o}C
hIGFBP4	CCTCTACATCATCCCCATCC	GGTCCACACACCAGCACTT	human	Tm = 56 ^{o}C
hPAPPA	AGCCAGCAGCATCCCAGGTGT	CGCCCGGAGCCAAAAAGTGGT	human	Tm = 62 ^{o}C
hSTC1	ATTCCCACCAACAAAATCCA	CCGTTCTAAAGGGATCCACA	human	Tm = 56 ^{o}C
hSTC2	ACTACTCAACTCTGCCGTCC	ACGCTTGGTTTCTTGGTGTC	human	Tm = 56 ^{o}C
mGAPDH	CCACTCACGGCAAATTCAACGGCA	ACCAGTAGACTCCACGACATACTC	mouse	Tm = 56 ^{o}C
mIGF1	CTGGTGGATGCTCTTCAGTTCG	TGCTTTTGTAGGCTTCAGTGGG	mouse	Tm = 58 ^{o}C
mIGF1R	GGAGAAGCCCATGTGTGAG	GTCGTGGATAACGAAGCCATC	mouse	Tm = 57 ^{o}C
mIGFBP4	GGAAAGGAATGGGGTGAGGA	AATATGGGGACGGAGGCAAA	mouse	Tm = 56 ^{o}C
mPAPPA	CACTTGGGCGGTATTGTCTT	TGGGTTGGTATCATTGCAGA	mouse	Tm = 56 ^{o}C
mSTC1	CCCAATCACTTCTCCAACAGA	GAAGAGGCTGGCCATGTTG	mouse	Tm = 56 ^{o}C
mSTC2	GACCCTCTGGAAGCAGTGAG	ACACATCCAGCGTGTGACAT	mouse	Tm = 60 ^{o}C

Table S6: Primers used for RT-qPCR analysis

Supplementary Methods

Tissue processing for LCM and gene expression analysis

Tissues were obtained from patients who had previously provided written, informed consent for sample collection to a Research Ethics Board- (REB) approved biobanking initiative (MUHC study identifiers SUR-99-780 and SUR-2000-966). 23 fresh frozen samples were micro-dissected into epithelium and stroma using a PixCell IIe LCM system (Arcturus) and RNA was then isolated (PicoPure RNA isolation kit, Arcturus) and concentrated (LabConco). RNA was amplified using the T-7 based RiboAmp HS Plus RNA amplification kit (Arcturus) and labelled with Cy3 dye (Turbo labeling kit Cy3, Life Technologies) for two-color microarray-based gene expression hybridization and scanning (Agilent). Raw data was normalized, log2-transformed, filtered, and quality control analysis carried out. HGNC gene annotations were applied and the mean was taken when multiple probes were present for the same gene. The batch effect was corrected using ComBat [1]. Gene expression of TS differentially expressed genes from our ILC-LCM dataset were applied to previously reported LCM-IDC (GSE68744) [2] and LCM-normal (GSE4823) [3] datasets, which comprise 32 IDC ER+ paired TE-TS samples and 58 normal breast paired epithelium-stroma samples respectively.

Pathway and network analysis

Reactome bioinformatics resources in R were used for biomolecular pathway annotation analysis. **Toppgene** (https://toppgene.cchmc.org/) pathway enrichment analysis was used to identify genes involved in pathways of interest (Benjamini–Hochberg adjusted p-value < 0.05). GeneMANIA functional network analysis using the Cytoscape 3.7.2 software was used to

identify human genes that were in the same pathway or were linked by known genetic or physical interactions. Nodes represent log2(FC TS/TE) expression as discrete mapping.

RNAScope

FFPE sections were obtained from the 23 ILC samples used for the generation of the LCM-ILC dataset. Each section was stained with a positive control probe Hs-PPIB to check the quality of the RNA. 20 out of 23 samples had good RNA integrity and were stained for human *PAPPA* (NM_002571.5) targeting 1274-2258. Expression of *PAPPA* was quantified in 6 x 1 mm^2 regions of interest per section using QuPath version 0.2.0, an open-source software for digital pathway image analysis (https://doi.org/10.1038/s41598-017-17204-5). The "cellular detection" function was used to segment the tumor and stroma in each region, with fibroblast morphology validated by a pathologist. Subsequently, subcellular detection was carried out to detect the number of dots per cell (each dot represents an mRNA transcript of the gene of interest). The number of dots per cell in the tumor and stroma were calculated. Statistical analysis was carried out using paired *t*-test.

Cell lines and generation of conditioned media

SUM44-PE (BioIVT), MDA-MB-134VI, MCF-7, T47D (all ATCC), KEP epithelial cells and fibroblasts (provided by Seth Coffelt, Cancer Research UK Beatson Institute, Glasgow) and primary human CAFs (isolated from tumors obtained via the NHS Lothian Tissue Governance Committee under approval 15/ES/0094) were grown at 37 ºC in humidified air incubators with 5% CO_2. All CAFs were seeded on collagen-coated plates using 1:30 PureCol (Advanced BioMatrix). Cells were authenticated via STR profiling at Culture Collections, UK using the Promega Powerplex 16 HS kit and confirmed to be mycoplasma-negative by qPCR performed at the MRC Human Genetics Unit, The University of Edinburgh. To collect conditioned media

(CM) cells were serum starved and the media collected after 24 and 48 hours (tumor cells) and 96 hours (CAFs). The CM was centrifuged at 4 ºC at 1600 rpm for 10 min to remove cell debris and filtered using a 0.22 μm filter.

PAPP-A ELISA

Conditioned media from SUM44-PE, MDA-MB-134VI, MCF-7, T47D, and primary ILC and IDC CAFs was used to detect levels of secreted PAPP-A in the media using a highly sensitive PAPP-A picoELISA kit as previously described [5]. PAPP-A concentration was normalized to total cell number. n = 3.

IGFBP-4 cleavage assay

Conditioned media from ILC and IDC primary CAFs was tested for PAPP-A ability to cleave IGFBP-4 as previously described [6]. Briefly, samples were incubated with radiolabeled (^{125}I) IGFBP-4 for 1, 2 and 4 hours. Cleavage products were separated by 10-20% SDS-PAGE and visualized by autoradiography. The degree of cleavage was quantified using a Typhoon imaging system (GE Healthcare), and background levels were subtracted. In some reactions, purified mAb1/41 antibody was added in known amounts as specified.

qRT-PCR

RNA was isolated from ILC and IDC cell lines and primary CAFs as well as from tumors isolated from the HCI-013 PDX model (a gift from Dr Alana Welm, Utah) using the RNeasy kit (Qiagen). RNA was converted into cDNA using the SuperScript First-Strand kit (Invitrogen). qPCR was performed with a SYBR Select Mastermix (Life Technologies) and the ΔΔCt was calculated as the relative expression to ED30 CAFs or KEP CAFs. n = 3. The

differences in gene expression were assessed by Mann-Whitney-Wilcoxon test and *t*-test. Primer sequences and melting temperatures (Tm) are summarised in Supplementary Table S6.

Immunofluorescence

Cells were grown on glass cover-slips and fixed with 4% paraformaldehyde for 10 min at room temperature. Fixed samples were stained for vimentin (1:200, 5741S, Cell Signaling Technologies), α-SMA (1:200, M0851, Dako), cytokeratin18 (1:200, 4548S, Cell Signaling Technologies), cytokeratin14 (1:200, in-house), estrogen receptor (1:200, ab16660, Abcam), PR (1:100, M3569, Dako), E-cadherin (1:100, 3195S, Cell Signaling Technologies), P-cadherin (1:150, AF761, R&D) overnight at 4 ºC. Alexa Fluor 488 and 549 (Invitrogen) secondary antibodies were applied for 45 min at room temperature. Cover-slips were attached to slides using Vectashield with DAPI (Vector Laboratories). Fluorescent images were captured using Olympus FV1000 confocal microscope and Fluoview software and processed using ImageJ (National Institutes of Health).

Immunofluorescence Staining of ILC tumors

Sections from paraffin-embedded ILC tumors were dewaxed in xylene, rehydrated through a graded concentration of ethanol. The sections then were washed with dH_20 and antigen retrieved by boiling in 10 mM citrate buffer (pH 6.0) for 5 mins. For permeabilization, the sections were washed with 0.05% PBST then blocked by with 2% bovine serum albumin in PBST for 1 hr at room temperature. The tissues were then incubated with PAPP-A (1:100, NBP1-90087) and alpha smooth muscle actin (Dako, 1:400) antibodies overnight at 4°C. The following day, the sections were washed with PBS and incubated with Alexa Fluor 594 goat anti-mouse IgG and Alexa Fluor 647 anti-rabbit secondary antibodies (1:200) for 1 hr at room temperature (in dark). The tissue sections were then washed three times with PBS followed by

one wash with dH_20. Once the sections were dried, they were mounted using NucBlue Reagent mounting medium (Invitrogen). Fluorescent images were captured using an Olympus FV1000 confocal microscope and processed using ImageJ.

References

1. Johnson WE, Li C, Rabinovic A. Adjusting batch effects in microarray expression data using empirical Bayes methods. Biostatistics 2007;8(1):118-27.
2. Oh EY, Christensen SM, Ghanta S, *et al.* Extensive rewiring of epithelial-stromal co-expression networks in breast cancer. Genome Biol 2015;16:128.
3. Finak G, Sadekova S, Pepin F, *et al.* Gene expression signatures of morphologically normal breast tissue identify basal-like tumors. Breast Cancer Res 2006;8(5):R58.
4. Pearce DA, Nirmal AJ, Freeman TC, *et al.* Continuous Biomarker Assessment by Exhaustive Survival Analysis. bioRxiv 2018;https://doi.org/10.1101/208660.
5. Gyrup C, Christiansen M, Oxvig C. Quantification of proteolytically active pregnancy-associated plasma protein-A with an assay based on quenched fluorescence. Clin Chem 2007;53(5):947-54.
6. Gyrup C, Oxvig C. Quantitative analysis of insulin-like growth factor-modulated proteolysis of insulin-like growth factor binding protein-4 and -5 by pregnancy-associated plasma protein-A. Biochemistry 2007;46(7):1972-80.
