# Supplementary figures and images for: Characterisation of the Stromal Microenvironment in Lobular Breast Cancer
Source: Cancers (Basel). 2022 Feb 11;14(4):904. doi: 10.3390/cancers14040904 (PMC8870100; doi:10.3390/cancers14040904)

Figure 6B

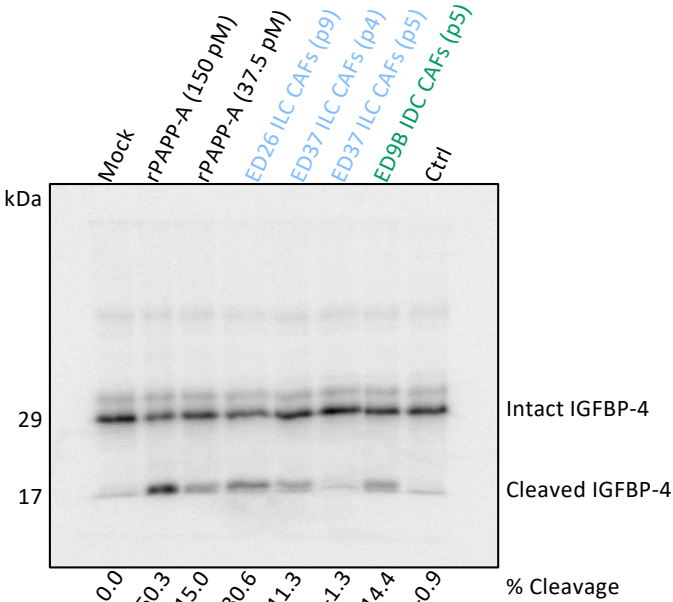

Figure 6c

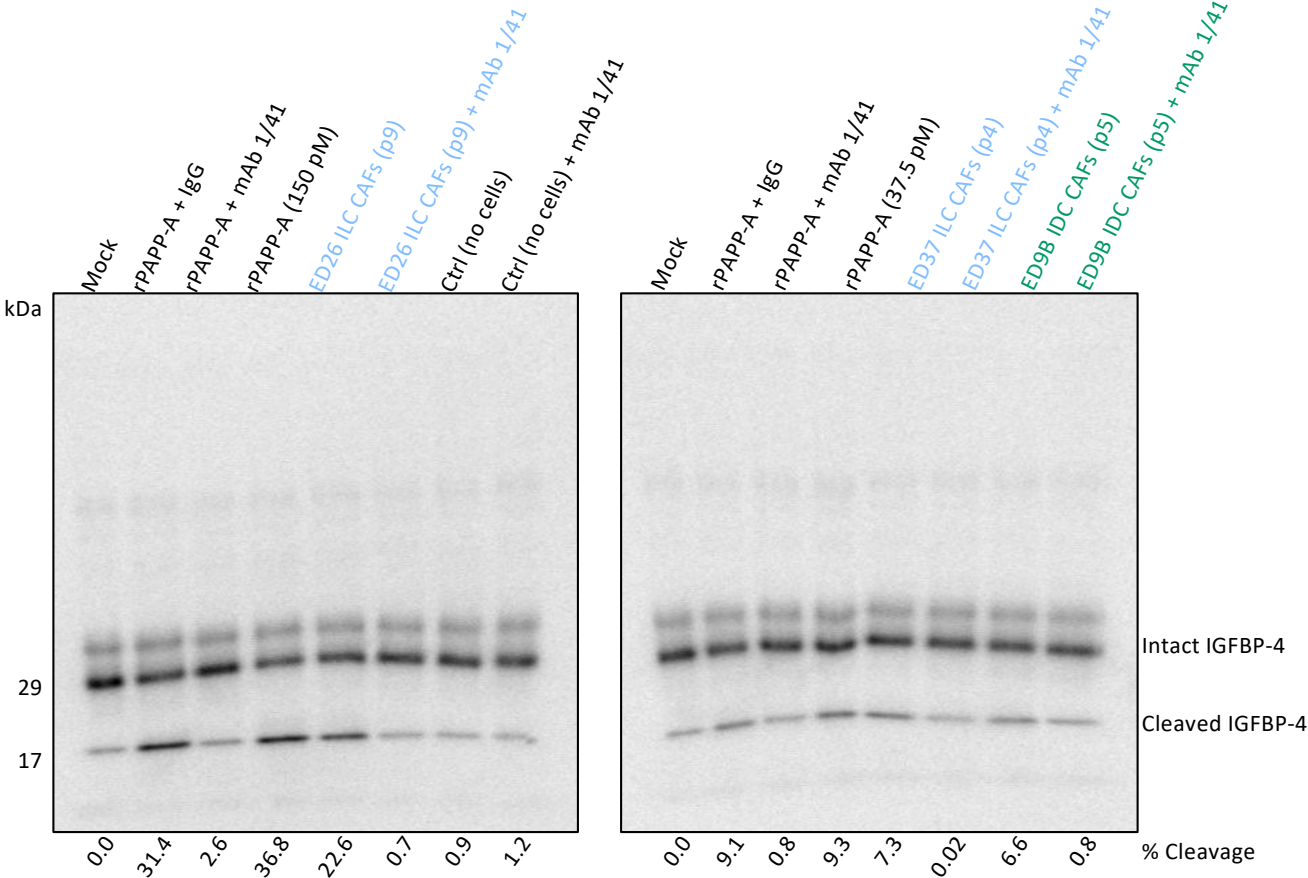

Supplement: Supplementary file 1 [file cancers-14-00904-s001.zip › Fig 6 full blots.pdf]
